# RPE-Targeted Laser Therapies for Central Serous Chorioretinopathy: Sublethal Modulation Versus Selective Injury and Repair—A Narrative Review

**DOI:** 10.3390/jcm15218600

**Published:** 2026-11-04

**Authors:** Seung Hee Jeon, Jeong Ah Shin, Young-Jung Roh

**Affiliations:** Department of Ophthalmology and Visual Science, Yeouido St. Mary’s Hospital, College of Medicine, The Catholic University of Korea, Seoul 07345, Republic of Korea; jsh881107@hanmail.net (S.H.J.); nole@daum.net (J.A.S.)

**Keywords:** central serous chorioretinopathy, nondamaging retinal laser therapy, retinal pigment epithelium, selective retina therapy, subthreshold micropulse laser

## Abstract

Central serous chorioretinopathy (CSC) is increasingly understood within the pachychoroid disease spectrum, with choroidal abnormalities playing a central pathogenic role. Nevertheless, the retinal pigment epithelium (RPE) remains critical to outer blood–retinal barrier function and subretinal fluid (SRF) homeostasis, and represents a potentially modifiable therapeutic target. RPE-targeted laser therapies employ biologically distinct approaches, ranging from sublethal RPE modulation with subthreshold micropulse laser and nondamaging retinal laser therapy to selective RPE injury followed by repair with selective retina therapy. We reviewed their laser–tissue interactions, dosimetry, clinical outcomes, and safety in CSC, focusing on how different RPE responses contribute to fluid resolution. Experimental and clinical observations provide a biological basis for considering how sublethal RPE modulation and selective RPE injury followed by repair may contribute to restoration of the RPE barrier and fluid homeostasis through distinct biological responses. Nevertheless, the mechanisms linking these RPE responses to SRF resolution remain incompletely understood. RPE-targeted laser therapies represent both potential therapeutic approaches and a means of gaining mechanistic insight into the contribution of RPE dysfunction to CSC. However, despite their distinct biological rationales, the clinical evidence supporting these modalities remains heterogeneous and insufficient to establish their comparative efficacy. Optimal treatment parameters, patient selection, and the long-term efficacy and safety of repeated treatment remain to be established.

## 1. Introduction

Central serous chorioretinopathy (CSC) is a chorioretinal disorder characterized by serous detachment of the neurosensory retina due to accumulation of subretinal fluid (SRF). It is often accompanied by retinal pigment epithelial (RPE) abnormalities or pigment epithelial detachment (PED). CSC predominantly affects working-age men, with an annual incidence of 9.9 per 100,000 in men and 1.7 per 100,000 in women reported in Olmsted County, Minnesota, USA [1]. Although its pathogenesis remains incompletely understood, contemporary concepts emphasize choroidal vascular hyperpermeability, venous congestion, and increased hydrostatic pressure within the pachychoroid spectrum, with RPE dysfunction representing a critical interface through which these abnormalities contribute to SRF accumulation [2,3].

Because persistent SRF may be associated with progressive photoreceptor and RPE alterations and visual dysfunction, SRF resolution is an important anatomical goal in CSC treatment [2,3]. Complete SRF resolution is thus commonly used as a key anatomical outcome in clinical studies evaluating treatment efficacy.

### 1.1. Evolution of Laser Therapy for CSC

Laser therapy has long been used in CSC management, reflecting the evolving understanding of the role of the RPE in the disease. Once fluorescein leakage in the CSC was recognized to originate at the RPE level, rather than from the retinal vasculature, focal laser photocoagulation was introduced to treat angiographically identified leakage sites [4,5]. Conventional continuous-wave laser photocoagulation, typically involving argon or diode lasers, was subsequently established as treatment for extrafoveal focal leakage [4,5,6].

By applying a visible thermal burn directly to the leakage site, conventional laser treatment could shorten the duration of SRF persistence; however, improvement in long-term visual acuity was not consistently achieved [4,5,6]. Notably, long-term follow-up of a randomized trial by Ficker et al. found that focal argon laser photocoagulation did not significantly reduce CSC recurrence or the development of chronic disease compared with observation [7]. Conventional photocoagulation poses a risk of collateral photothermal damage to the RPE, photoreceptors, and adjacent chorioretinal tissue, which is associated with potential complications, including scotoma, reduced contrast sensitivity, progressive laser scars, and secondary macular neovascularization. This substantially limits the application of conventional photocoagulation for subfoveal or juxtafoveal leakage sites [6,8].

This limitation prompted the development of tissue-sparing laser strategies designed to target the RPE while minimizing damage to the overlying neurosensory retina. Several approaches subsequently emerged, including subthreshold micropulse laser (SMPL) [9,10,11], nondamaging retinal laser therapy (NRT) [12,13,14], and selective retina therapy (SRT) [15,16,17]. Although these modalities all aim to avoid conventional chorioretinal photocoagulation, they involve substantially different laser–tissue interactions and biological endpoints. SMPL and NRT are based on sublethal thermal modulation without intentionally producing visible retinal damage [10,11,12,13], whereas SRT intentionally induces selective RPE injury, which is followed by cellular repair and repopulation [15,16,17].

In parallel, increasing recognition of choroidal vascular abnormalities, including choroidal dilation, hyperpermeability, and pachychoroid-related structural changes, has shifted therapeutic attention toward the choroid [2,18]. Verteporfin photodynamic therapy (PDT), particularly reduced-dose or reduced-fluence protocols, has increasingly been used for chronic CSC. Randomized clinical trials subsequently provided stronger evidence favoring PDT over specific comparator treatments, although the overall certainty of comparative evidence remains limited [3,19]. The PLACE trial, in particular, demonstrated that half-dose PDT had greater efficacy than did high-density subthreshold micropulse laser [20]. Similarly, the SPECTRA trial demonstrated superior short-term efficacy of half-dose PDT compared with oral eplerenone in chronic CSC [21]. However, the 2025 Cochrane network meta-analysis highlighted the limitations and uncertainty of the available comparative evidence and did not establish the overall superiority of any intervention [22]. While this choroid-centered perspective has established PDT as the preferred treatment for chronic CSC [3,19], it does not diminish the potential importance of the RPE as a therapeutic target.

### 1.2. The RPE as a Therapeutic Target in Contemporary CSC

The clinical expression of CSC ultimately involves disruption of fluid homeostasis across the RPE. The RPE constitutes the outer component of the blood–retinal barrier and regulates fluid transport from the subretinal space toward the choroid [23,24,25]. Alteration of normal RPE fluid transport, including a proposed reversal of transport polarity, has also been suggested as a potential contributor to SRF accumulation in CSC [23]. Fluorescein angiography (FA) has shown focal or multifocal leakage at the RPE level, while serous PED and other RPE abnormalities frequently accompany active disease [2,3]. Even within contemporary choroid-centered models, the RPE is understood to be a critical interface through which abnormal choroidal fluid may enter and accumulate in the subretinal space [18,23,25].

The spatial distribution of choroidal vascular hyperpermeability does not necessarily correspond to the distribution of RPE leakage seen on FA [26,27]. Choroidal hyperpermeability may involve multifocal or broad areas of the posterior pole, whereas fluorescein leakage may remain confined to one or a few focal RPE sites [27]. This spatial mismatch implies that choroidal abnormalities alone might not fully determine the location of fluid leakage and that regional differences in RPE susceptibility or functional reserve may contribute to CSC clinical expression [18,28]. This interpretation is also compatible with a choroid-first model, in which leakage may preferentially occur at sites where local RPE functional reserve is insufficient to withstand the increased choroidal fluid load.

This mismatch, along with the historically demonstrated efficacy of focal photocoagulation at angiographic leakage sites [4,5], further supports targeting the RPE as a site for direct therapeutic intervention. The development of tissue-sparing laser approaches has extended this approach by selectively targeting the RPE while minimizing collateral damage to the surrounding retina [8,11,17].

An RPE-targeted perspective should be viewed as complementary to contemporary pachychoroid-based concepts, rather than as an alternative to them. The choroid and RPE may represent interacting components of the same fluid-homeostatic pathway, with their relative contributions varying across patients and disease states. RPE-directed interventions may therefore provide a complementary therapeutic approach while also offering insights into how focal or diffuse RPE dysfunction, barrier integrity, and regenerative capacity contribute to the clinical expression of CSC.

### 1.3. RPE-Targeted Laser Approaches in CSC

RPE-targeted laser therapy comprises several modalities with markedly different laser–tissue interactions. SMPL was developed to reduce collateral retinal damage by dividing laser emission into short, repeated pulses that are separated by cooling intervals [10,11]. At sufficiently low duty cycles and energy settings, this approach is intended to induce a sublethal thermal response in the RPE without producing a visible burn. The proposed biological effect is therefore principally one of cellular modulation, rather than tissue destruction [10,11]. Treatment protocols, however, vary considerably in terms of wavelength, duty cycle, energy setting, and treatment area [9,10,11].

A related but technically distinct approach is NRT, which uses continuous-wave photothermal exposure. In contrast to micropulse delivery, continuous-wave laser exposure can be applied within a sublethal thermal range, below the threshold that results in detectable retinal injury [12,13]. Model-based dosimetry systems, such as Endpoint Management (EpM), were subsequently developed to provide more predictable control of laser exposure within this nondamaging treatment range [13]. This approach has also been applied clinically in CSC [14].

SRT differs fundamentally from both SMPL and NRT. SRT uses microsecond laser pulses, typically 30 pulses of 1.7 μs duration delivered at 100 Hz in classical clinical protocols, to injure RPE cells selectively while minimizing thermal diffusion to the surrounding neurosensory retina and choroid [15,16,17,29]. The response to this treatment involves migration and proliferation of neighboring RPE cells and restoration of the treated RPE monolayer [15,16,17]. Whereas subthreshold approaches primarily seek to preserve treated RPE cells and induce cellular modulation by sublethal means, SRT deliberately crosses the threshold for selective RPE cellular injury, while minimizing collateral damage to the overlying neurosensory retina [15,16,17,29].

### 1.4. Scope of the Review

This review focuses on RPE-targeted laser interventions used for treating CSC, with particular emphasis on SMPL, NRT, and SRT. These three modalities were selected because they provide a basis for comparing different RPE-targeted mechanisms, treatment methodologies, and clinical outcomes in CSC. Other RPE-targeted laser approaches, including nanosecond laser therapy, are recognized as mechanistically relevant but are not specifically reviewed, as the present review is primarily structured around the CSC-specific clinical evidence available for the three selected modalities. Conventional focal photocoagulation is discussed mainly as the historical foundation of RPE-directed treatment and as a reference point for understanding the transition from destructive photocoagulation toward tissue-sparing and selective RPE-targeted strategies.

We examine the biological basis and laser–tissue interactions of these modalities, differences in dosimetry and treatment methodology, and the available clinical evidence regarding anatomical efficacy, visual outcomes, retreatment requirements, and safety. Particular attention is given to the distinction between sublethal RPE modulation and selective RPE injury followed by repopulation, because these approaches are frequently grouped together in discussions, despite their fundamentally different biological endpoints.

SRT is discussed in greater mechanistic detail because its experimentally characterized selective RPE injury and subsequent reparative response provide a useful model for examining the potential contribution of localized RPE repair to CSC treatment. This emphasis does not imply greater clinical efficacy or a higher level of clinical evidence for SRT than for SMPL or NRT. Sublethal RPE modulation, including cellular stress responses and associated molecular changes, represents a complementary mechanistic approach and is also considered in this review. Clinical responses across different CSC phenotypes are reviewed where evidence is available, with the proposed biological mechanisms evaluated separately from the strength of the supporting clinical evidence.

PDT is not reviewed comprehensively as a primary therapeutic modality because extensive evidence-based reviews and guidelines have already addressed its efficacy and position in CSC management [3,19]. Instead, PDT is discussed where necessary as a choroid-directed comparator treatment for interpreting the clinical role of RPE-targeted laser therapy.

Current evidence-based assessments indicate that the clinical evidence supporting SMPL and SRT remains less established than that supporting PDT. Feenstra et al. [3] concluded that SMPL was less effective than half-dose PDT in achieving complete SRF resolution in chronic CSC and that the evidence supporting SRT remained limited, with further comparative studies needed. Similarly, the 2025 American Academy of Ophthalmology assessment [19] classified the randomized PLACE trial comparing SMPL with half-dose PDT as Level II evidence, favoring PDT for short-term anatomical outcomes. The assessment also classified a small randomized SRT trial as Level II evidence, although its limited sample size precluded definitive conclusions regarding clinical efficacy. Thus, despite their distinct biological approaches and encouraging clinical findings, the comparative effectiveness of SMPL and SRT requires further investigation.

The overall aim of this review is not to propose an alternative to contemporary pachychoroid-centered concepts, but to define the biological and clinical significance of RPE-targeted laser therapy within the broader pathophysiological spectrum of CSC and to consider how different forms of RPE intervention may contribute to future mechanism-based and individualized treatment strategies.

## 2. Literature Search

PubMed/MEDLINE was searched from 1 July to 21 July 2026 to identify studies relevant to RPE-targeted laser therapy for CSC. No publication date restrictions were applied in this review. Search terms included combinations of “central serous chorioretinopathy,” “subthreshold micropulse laser,” “micropulse laser,” “subthreshold diode micropulse,” “subthreshold diode micropulse laser,” “nondamaging retinal therapy,” “nondamaging retinal laser,” “selective retina therapy,” “selective retinal therapy,” “nanosecond laser,” and “retinal rejuvenation therapy.” The reference lists of relevant articles and reviews were additionally screened to identify further pertinent experimental and clinical studies. We included original clinical studies, experimental studies, and relevant review or consensus articles when they provided information on treatment mechanisms, dosimetry and treatment methodology, clinical outcomes, or safety. Reports with insufficient methodological information or without direct relevance to RPE-targeted laser mechanisms or CSC treatment were excluded. Table 1 was specifically designed to summarize selected randomized controlled trials evaluating clinical outcomes of RPE-targeted laser therapies in CSC, rather than the full range of studies included in this narrative review. No geographic restriction was applied to the literature. Given the narrative scope of this review, studies were selected based on their relevance to the topic rather than according to a predefined systematic-review protocol. The search was limited to PubMed/MEDLINE, supplemented by reference list screening. The numbers of retrieved and screened records were not systematically documented, which limits the reproducibility and assessment of the completeness of the literature search.

For consistency, varying terminology used in the literature for SMPL treatment was standardized to SMPL throughout this review.

## 3. Biological Basis of RPE-Targeted Laser Therapy

### 3.1. RPE Barrier and Fluid Transport in CSC

The RPE makes up the outer blood–retinal barrier. It actively maintains SRF homeostasis by transporting fluid and ions from the subretinal space toward the choroid [23,24,25]. In CSC, disruption of this barrier and transport function represents the final interface through which abnormalities originating from the choroid translate into SRF accumulation [2,18]. Although the precise contribution of primary RPE dysfunction remains uncertain, RPE abnormalities in CSC commonly occur in the setting of more widespread choroidal dysfunction, including choroidal congestion, hyperpermeability, and increased hydrostatic pressure [2,3,18].

**Figure 1:**
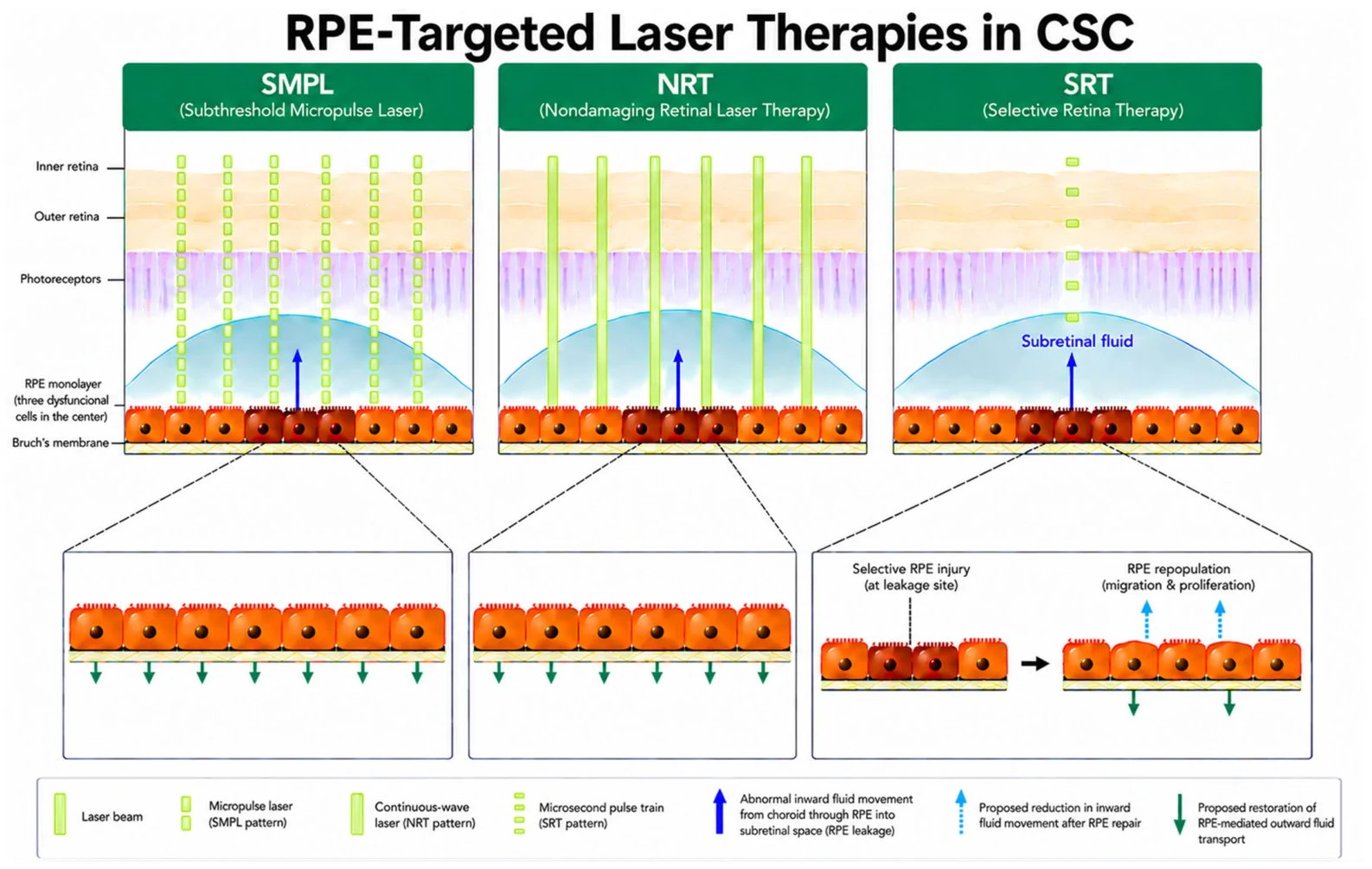
Conceptual illustration of RPE-targeted laser therapies in central serous chorioretinopathy (CSC).

FA has shown that this disturbance may be expressed as focal, multifocal, or more diffuse leakage at the RPE level [2,3], although the extent of choroidal abnormality and the pattern of RPE leakage do not necessarily coincide spatially [26,27]. Choroidal abnormalities may extend beyond the sites of angiographically detectable RPE leakage [26,27]. The development and persistence of SRF may therefore reflect an interaction between choroidal fluid load and the local capacity of the RPE to maintain barrier integrity and fluid clearance [18,23,24,25]. Regardless of whether RPE dysfunction is primary or secondary to choroidal abnormalities, loss of local barrier integrity may facilitate fluid entry into the subretinal space, while impaired RPE fluid transport may further contribute to its persistence [23,24,25], leaving the RPE as a critical therapeutic interface regulating SRF accumulation and clearance. These findings provide a biological rationale for RPE-targeted laser approaches [7,13,14,15,16], as conceptually illustrated in Figure 1.

Subthreshold micropulse laser (SMPL), nondamaging retinal laser therapy (NRT), and selective retina therapy (SRT) involve distinct laser–tissue interactions and treatment strategies. The schematic depicts focal retinal pigment epithelium (RPE) leakage with associated subretinal fluid. SMPL and NRT are illustrated as broader-area treatments, whereas SRT is directed toward angiographically identified leakage areas. SRT targeting occurs at the laser-spot level, not at the individual-cell level; a typical 200-μm spot encompasses numerous RPE cells, and angiographic localization is not precise at the cellular level. Treatment areas may be broader in cases of diffuse leakage. SMPL and NRT aim to stimulate RPE function without causing clinically apparent tissue damage, potentially enhancing RPE-mediated outward fluid transport. In contrast, SRT induces selective RPE injury followed by migration and proliferation of surrounding RPE cells, promoting RPE repair and potentially restoring local barrier function and outward fluid transport while reducing abnormal inward leakage. The illustrated fluid movements and therapeutic responses represent proposed mechanisms rather than established effects. Because laser spot sizes differ among modalities and each spot encompasses numerous RPE cells, laser spot dimensions, pulse patterns, treatment distributions, and cellular representations are schematic and not to scale.

### 3.2. SMPL: Sublethal RPE Modulation

SMPL represents an RPE-targeted strategy designed to induce a biological response without intentionally causing RPE cell death. Unlike conventional continuous-wave photocoagulation, in which sustained thermal exposure produces coagulative tissue injury, micropulse delivery divides the laser energy into brief, repetitive exposures separated by “off” periods. These intervals allow partial thermal relaxation between pulses and limit cumulative temperature elevation within the RPE and adjacent retina. When delivered below the threshold for causing detectable tissue injury, this pattern is intended to generate sublethal thermal stress, while preserving RPE cellular integrity. The absence of visible laser-induced retinal damage after treatment is a defining feature of the subthreshold micropulse treatment concept [8,9,10,11,30].

The proposed biological effect of SMPL is based primarily on cellular stress modulation, rather than tissue destruction and subsequent wound repair. Sublethal thermal stress has been proposed to activate heat-shock proteins (HSPs) and other cellular stress-response pathways that contribute to protein repair, cellular homeostasis, and recovery from metabolic stress [8,10,11,30]. Experimental studies have demonstrated biological responses to SMPL in the absence of conventional photocoagulative injury, while molecular analyses have identified changes in gene expression following SMPL that differ from those induced by continuous-wave laser irradiation [30,31]. These findings support the concept that SMPL can induce cellular responses without causing histologically detectable retinal injury.

Beyond HSP-mediated stress responses in the RPE, other cellular pathways may also contribute to the biological effects of SMPL [10]. In an experimental mouse study, De Cillà et al. demonstrated that SMPL modulated oxidative stress, antioxidant defenses, apoptosis, and autophagy-related pathways [32]. Furthermore, Midena et al. reported reductions in Müller cell-associated biomarkers, including glial fibrillary acidic protein (GFAP) and Kir4.1, together with decreased vascular endothelial growth factor (VEGF) levels following SMPL treatment in patients with diabetic macular edema [33]. These findings suggest that SMPL-induced biological responses may extend beyond RPE stress modulation to involve retinal glial activity and associated molecular signaling pathways. However, because much of this evidence derives from experimental models or studies of diabetic retinal disease, its relevance to the therapeutic mechanisms of SMPL in CSC remains to be established [10,32,33].

In the RPE, these responses have been hypothesized to improve the ability of stressed but viable cells to maintain retinal homeostasis. The therapeutic effect of SMPL has been interpreted primarily as functional modulation of existing RPE cells, rather than replacement of damaged RPE through proliferation and migration [8,10,11], but the precise molecular pathways responsible for its clinical effects remain incompletely established. Much of this mechanistic interpretation is based on experimental observations and biological inference rather than on direct demonstration in human CSC [30,31].

Because SMPL treatment is intended to preserve rather than destroy irradiated RPE cells, broader and denser treatment has been proposed to increase the effective RPE surface area exposed to sublethal stimulation [34]. In a clinical series of CSC cases, several hundred micropulse applications were delivered over areas of RPE leakage and pigmentary abnormality, and the results provided clinical support for the feasibility of this high-density treatment concept [34]. However, the relationship between treatment density and specific molecular responses, including HSP expression, has not been directly established.

### 3.3. NRT: Controlled Sublethal Photothermal Stimulation

NRT represents a sublethal photothermal approach designed to induce a biological response in the RPE without intentionally causing cellular destruction. In the clinical approach discussed in this review, NRT is performed using a 577-nm PASCAL laser system (Topcon Medical Laser Systems, Santa Clara, CA, USA) with EpM, which adjusts laser power and exposure duration according to a predefined percentage of an individually determined visible-lesion threshold. Exposure durations may vary according to the treatment protocol, with 15- and 20-ms exposures reported in clinical and preclinical studies, respectively [35]. Unlike SMPL, which limits thermal accumulation through repetitive short exposures separated by cooling intervals, NRT controls the magnitude of photothermal exposure relative to an individualized tissue-damage threshold [10,11,13].

The biological rationale for NRT rests on the existence of a therapeutic window in which controlled thermal stimulation may induce cellular stress responses without producing irreversible RPE injury [12,13,35]. EpM was developed to provide reproducible dosimetry within this range by using a computational model of retinal photothermal damage [13]. NRT and SMPL therefore share the objective of sublethal RPE modulation, but differ in the method used to control thermal exposure. Clinical application of NRT using a PASCAL laser with EpM has been reported in CSC [14]. The relationship between the delivered thermal dose, cellular response, and clinical efficacy, however, requires further study.

### 3.4. SRT: Selective RPE Injury, Repair, and Repopulation

SRT represents a distinct RPE-targeted laser strategy in which injury is intentionally and selectively induced in RPE cells, while damage to the surrounding neurosensory retina and choroid is minimized. Selective targeting is based on the preferential absorption of visible laser energy by melanosomes within RPE cells. Experimental and clinical SRT systems have used green wavelengths, around 527 nm, and pulse durations of approximately 2 µs or less, allowing rapid energy deposition in melanosomes while limiting thermal diffusion to the adjacent retinal structures [15,16,17,29,36]. Experimental studies using 527-nm Nd:YLF (neodymium-doped yttrium lithium fluoride) irradiation, including 1.7-µs and shorter pulse durations, have demonstrated selective RPE injury with relative preservation of the photoreceptors and other retinal structures. Shorter pulse durations reduce heat diffusion from melanosomes and influence both the RPE damage threshold and the therapeutic window [29,36].

At appropriate pulse energies, rapid heating of melanosomes can result in transient microbubble formation around these intracellular absorbers. The expansion and subsequent collapse of these short-lived microbubbles mechanically disrupt RPE cells, producing localized cellular injury, without the broader thermal damage characteristic of conventional continuous-wave photocoagulation [16,29]. The desired SRT effect therefore occurs within a therapeutic window in which RPE cellular injury is achieved while collateral injury to the photoreceptors, neural retina, and choroid is minimized. This therapeutic window is influenced by pulse duration, pulse number, and tissue pigmentation, which highlights the importance of appropriate dosimetry [29,36].

Selective RPE injury is followed by a wound-healing response involving the surrounding viable RPE. Experimental studies have demonstrated migration, remodeling, and proliferation of RPE cells neighboring the targeted site, followed by repopulation of the treated area and restoration of the RPE monolayer, while largely preserving the adjacent photoreceptor layer [15,16,17]. This reparative response forms the biological basis for the concept of RPE repair and repopulation after SRT. However, the precise spatial extent and reproducibility of this reparative response have not been established. Accordingly, clinical SRT studies have generally targeted angiographically identified leakage areas rather than defining treatment coverage based on a predetermined radius of RPE remodeling [8,17].

## 4. Laser–Tissue Interactions and Treatment Methodology

### 4.1. SMPL

Clinical SMPL studies of CSC have predominantly used 577-nm yellow or 810-nm infrared lasers, with considerable variation in power, exposure duration, spot size, and duty cycle. Duty cycles ranging from approximately 5% to 15% have been reported, with lower duty cycles allowing greater thermal relaxation between micropulses. Therapeutic settings result in no clinically detectable retinal lesion [9,10,11].

The marked variation in treatment parameters and spatial patterns among clinical studies is summarized in Table 1. Some protocols have targeted angiographically identified leakage sites, whereas others have used high-density or confluent treatment over broader angiographically defined treatment areas. In one randomized study, grid treatment was applied over the total area of SRF. Treatment may therefore range from relatively focal leakage-directed application to denser treatment covering a broader affected area. Because SMPL is intended to modulate the viable RPE, rather than induce selective RPE injury at an individual leakage site, the biological dose may depend not only on the energy delivered per spot, but also on the density and spatial extent of the treatment.

**Table 1:** Randomized controlled trials of subthreshold micropulse laser for central serous chorioretinopathy.

Study [Reference]	Design	Study Population	Laser Parameters/Dosimetry	Treatment Strategy	Anatomical Outcome (SRF Resolution)	Functional Outcomes	Follow-Up	Retreatment Permitted Before Outcome Time Point (Yes/No, When)	Masking of Outcome Assessor
Kretz et al. (2015) [37]	Randomized controlled trial	Persistent CSC > 3 months; 20 SMPL vs. 24 half-dose PDT vs. 18 observation	810 nm; 75–125 μm; 300 ms; 15% duty cycle; initial power 1000 mW	FA-guided, applied to active leakage site; mean 101 spots	Not separately reported; absence of persistent FA leakage in 60% at 16 weeks	BCVA + 6.7 ETDRS letters	16 weeks	Yes; at 8 weeks and thereafter, as needed	Yes; masked photographer and retina specialist
van Dijk et al. (2018) [20]	Multicenter randomized controlled trial	Chronic CSC; 90 SMPL vs. 89 half-dose PDT	810 nm; 125 μm; 200 ms; 5% duty cycle; ~1800 mW	ICGA-guided; high-density treatment; mean 187 spots	Complete SRF resolution: 6–8 weeks (before retreatment): 13.8% (11/80) 7–8 months (retreatment permitted): 28.8% (19/66)	BCVA change: +1.39 ETDRS letters at 6–8 weeks; +4.48 letters at 7–8 months. Retinal sensitivity change: +0.92 dB at 6–8 weeks; +1.38 dB at 7–8 months.	7–8 months	6–8 weeks: No (before retreatment); 7–8 months: Yes (retreatment at 6–8 weeks for persistent SRF).	Not reported (open-label trial).
Arora et al. (2019) [38]	Randomized controlled trial	Acute CSC (<2 months); 34 SMPL vs. 34 observation	810 nm; 125 μm; 200 ms (100 micropulses); 15% duty cycle; 2× threshold power	Leakage-directed; ~100 treatment envelopes applied to the leakage point	Persistent/recurrent SRF at 6 months: 11.8% (4/34) in the SMPL group vs. 29.4% (10/34) in the observation group (*p* = 0.036).	BCVA (logMAR): 0.59 at baseline, 0.06 at 8 weeks, and 0.03 at 6 months (SMPL group); significantly better than observation at 8 weeks (*p* < 0.001) and 6 months (*p* = 0.008).	6 months	Not reported during the 6-month follow-up	Not reported
Sun et al. (2020) [39]	Prospective randomized double-masked non-inferiority trial	CSC; 44 SMPL vs. 44 conventional laser	577 nm; 160 μm; 200 ms; 5% duty cycle; 50% of threshold power	FA-guided high-density treatment; 150–200 confluent spots	Complete SRF resolution at 12 weeks: 63.6% (28/44) vs. 81.8% (36/44) (SMPL vs. TCL; *p* = 0.056)	BCVA gain at 12 weeks (SMPL vs. TCL): FAS, +6.23 ± 8.59 vs. +6.61 ± 6.35 ETDRS letters; ITT, +5.55 ± 8.45 vs. +5.45 ± 5.26 letters	12 weeks	No; retreatment assessed at week 12	Yes; masked optometrist and OCT technicians
Zhou et al. (2021) [40]	Single-center randomized controlled trial	Acute CSC; 55 SMPL vs. 55 conventional laser	577 nm; 100 μm; 200 ms; 5% duty cycle; 50% of threshold power (400–600 mW)	Dense treatment over leakage points; <50 spots	Complete SRF resolution (ITT; SMPL vs. CL): 72.7% (40/55) vs. 89.1% (49/55) at 3 months (*p* = 0.029); 85.5% (47/55) vs. 92.7% (51/55) at 6 months (*p* = 0.221).	BCVA (logMAR; SMPL vs. CL): baseline, 0.32 vs. 0.39; 3 months, 0.03 vs. 0.01; 6 months, 0.00 vs. 0.00.	6 months	3 months: No; 6 months: Yes (at 3 months for persistent SRF).	Not reported.
Zhou et al. (2024) [41]	Prospective open-label randomized non-inferiority trial	Acute CSC; 34 SMPL vs. 34 half-dose PDT	577 nm; 100 μm; 200 ms; 5% duty cycle; 50% of visible threshold; treatment power 400–600 mW	Dense treatment over FA leakage; ≤50 spots/session	Complete SRF resolution at 6 months (ITT; SMPL vs. half-dose PDT): 82.4% (28/34) vs. 91.2% (31/34); *p* = 0.3724.	BCVA gain at 6 months (SMPL vs. half-dose PDT): ITT, +12.85 ± 10.65 vs. +13.26 ± 10.38 ETDRS letters; per-protocol, +12.64 ± 10.74 vs. +13.52 ± 10.44 letters.	6 months	Not reported.	Not reported (open-label trial).
Brelen et al. (2024) [42]; Ho et al. (2021) [43], interim OCTA analysis	Prospective double-masked randomized controlled non-inferiority trial; interim OCTA analysis of 33 participants	Chronic CSC (>3 months); full cohort: 34 SMPL vs. 34 half-dose PDT; interim OCTA subgroup: 18 vs. 15	577 nm; 200 μm; 200 ms; 5% duty cycle; 320–360 mW (full cohort); 340–400 mW reported in interim analysis	Grid treatment over the SRF area; 0 spacing between spots	Full cohort: Complete SRF resolution (SMPL vs. half-dose PDT): 24.2% (8/33) vs. 58.8% (20/34) at 1 month; 46.9% (15/32) vs. 70.6% (24/34) at 3 months; 82.1% (23/28) vs. 90.9% (30/33) at 12 months. Interim OCTA analysis: Complete SRF resolution at 3 months: 50.0% (9/18) vs. 86.7% (13/15).	Full cohort: BCVA change at 12 months (SMPL vs. half-dose PDT): −0.12 ± 0.21 vs. −0.13 ± 0.12 logMAR; retinal sensitivity change: +5.70 ± 5.02 vs. +6.05 ± 3.83 dB. Interim OCTA analysis: BCVA at 6 months: 0.11 ± 0.06 vs. 0.09 ± 0.06 logMAR.	12 months (full cohort); 6 months (interim OCTA analysis)	Full cohort: 1 and 3 months: No; 12 months: Yes (retreatment at months 3, 6, and 9; rescue PDT permitted). Interim OCTA analysis: 3 months: No; 6 months: Yes (retreatment every 3 months for persistent SRF).	Full cohort: Yes; masked assessors and optometrists. Interim OCTA analysis: Yes; masked investigators.

Abbreviations: BCVA, best-corrected visual acuity; CL, conventional laser; CSC, central serous chorioretinopathy; ETDRS, Early Treatment Diabetic Retinopathy Study; FA, fluorescein angiography; FAS, full analysis set; ICGA, indocyanine green angiography; ITT, intention-to-treat; logMAR, logarithm of the minimum angle of resolution; OCT, optical coherence tomography; OCTA, optical coherence tomography angiography; PDT, photodynamic therapy; SMPL, subthreshold micropulse laser; SRF, subretinal fluid; TCL, threshold conventional laser. Note: Laser parameters are reported as used in the original studies. Although earlier studies by Kretz et al. and Arora et al. employed a 15% duty cycle, a 5% duty cycle has been more commonly adopted in recent clinical protocols and is recommended in contemporary SMPL treatment guidance to reduce the risk of thermal retinal injury [10].

Dosimetry remains a major methodological issue with SMPL because the therapeutic exposure produces no directly observable endpoint. In titration-based protocols, a visible or barely visible threshold is first determined at a test location, after which the power used for treatment is reduced to a predefined fraction of that threshold. However, both threshold-based titration and standardized fixed-parameter strategies have been used in previous studies [10,11], contributing to the methodological heterogeneity across clinical studies of CSC (Table 1). In fixed-power protocols, such as the 1800-mW setting used in the PLACE trial, interindividual differences in retinal pigmentation and laser absorption may result in insufficient therapeutic exposure in some eyes, potentially contributing to differences in outcomes across studies [20].

Wavelength, power, duty cycle, exposure duration, spot size, retinal pigmentation, and treatment density can all influence effective tissue exposure; however, the biological effect at an individual treatment site cannot be directly confirmed during treatment. This lack of an observable lesion-specific endpoint remains an important challenge for standardization and comparison of SMPL protocols.

### 4.2. NRT

NRT treatment using a 577-nm PASCAL laser with EpM is based on individualized threshold titration. The power required to create a barely visible test lesion is first established as the 100% reference endpoint, after which EpM algorithmically reduces laser exposure to a predefined subvisible treatment level. In clinical studies of chronic CSC, treatment has typically been delivered at approximately 30% of the individually determined threshold, with no clinically visible lesion left at the actual treatment sites [13,14].

In reports of clinical applications, treatment has been delivered in a patterned manner over the involved macular region, rather than as conventional photocoagulation of individual leakage points [14,44]. Because the therapeutic endpoint itself is not directly visible, treatment relies on the accuracy of the initial threshold determination and the EpM dosimetry model used. Individual titration is important because pigmentation and other ocular characteristics can influence laser absorption and the visible-damage threshold. Although EpM provides a systematic method for translating an individualized visible-damage threshold into a subvisible treatment setting, the actual biological effect at individual treatment sites cannot be directly confirmed during treatment [13,14].

### 4.3. SRT

In SRT, because the intentionally created lesion is ophthalmoscopically invisible immediately after irradiation, but can be detected as RPE leakage on FA, individualized titration has traditionally been used to confirm selective RPE injury. Laser absorption and the therapeutic window vary with individual RPE pigmentation and other tissue characteristics. Accordingly, a single fixed pulse energy cannot be uniformly applied across eyes, and individualized dosimetry or titration is an important aspect of SRT. Consistent with this principle, prospective clinical studies have used different dosimetry approaches and reported variable pulse energies, reflecting differences in treatment protocols and energy-selection methods (Table 2). Traditionally, conventional titration uses test spots reflecting progressively increasing pulse energy outside of the treatment area to identify an exposure that produces FA-visible RPE leakage, without an immediately ophthalmoscopically visible retinal lesion [15,16,17].

**Table 2:** Prospective clinical studies of FA leakage-directed selective retina therapy for central serous chorioretinopathy.

Study [Reference]	Design	Study Population	Dosimetry/Laser Parameters	Treatment Pulse Energy	Complete SRF Resolution	Functional Outcomes	Follow-Up	Retreatment Permitted Before Outcome Time Point (Yes/No, When)	Masking of Outcome Assessor
Klatt et al. (2011) [45]	Prospective randomized controlled trial	Symptomatic CSC with ≥3 months of reduced BCVA; 14 SRT/16 untreated controls	Optoacoustic feedback-guided dosimetry; 527-nm Nd:YLF laser; 200-μm spot; 1.7-μs pulse duration; 30 pulses/spot; 100 Hz.	Mean 168.6 ± 31 μJ (range, 120–270 μJ)	Complete SRF resolution at 3 months: 71.4% vs. 40.0% (6/16) (SRT vs. observation; *p* = 0.081).	BCVA gain at 3 months: +12.6 ± 7.2 vs. +6.3 ± 8.9 ETDRS letters (SRT vs. observation; *p* = 0.043).	3 months	No; control-group crossover to SRT after the 3-month assessment.	Yes; masked BCVA examiner.
Framme et al. (2015) [46]	Prospective two-center study	Acute CSC: 10 eyes; chronic-recurrent CSC: 16 eyes	FA-guided individualized titration (optoacoustic feedback for slit-lamp delivery); 527-nm Nd:YLF laser; 200-μm spot (slit-lamp delivery); 300-ns or 1.7-μs pulse duration; 30 pulses/spot; 100 Hz.	200–250 μJ (1.7 μs) and 100–180 μJ (300 ns)	Acute CSC: Complete SRF resolution: 50.0% (5/10) at 4 weeks and 100% (10/10) at 3 months. Chronic-recurrent CSC: 18.8% (3/16) at 3 months after initial SRT; 83.3% (5/6) after repeat SRT (median 25 days).	Acute CSC: Mean BCVA increased from 76.6 to 77.9 ETDRS letters at 4 weeks and 85.0 letters at 3 months. Chronic-recurrent CSC: Mean BCVA increased from 71.6 to 72.8 ETDRS letters at 3 months after initial SRT.	3 months; longer after re-SRT	Acute CSC: No. Chronic-recurrent CSC: Initial 3-month outcome: No; repeat SRT performed at 1–5 months for persistent SRF/leakage.	Not reported.
Oh et al. (2021) [47]	Randomized sham-controlled clinical trial	Idiopathic CSC with symptoms for ≥3 months; 31 SRT/37 sham controls	FA-guided individualized titration using optoacoustic and reflectometry real-time feedback; 527-nm Nd:YLF laser; 1.7-μs pulse duration; 100 Hz; pulse energy range, 30–350 μJ; spot size, and pulses per spot not reported.	Not reported.	Complete SRF resolution (SRT vs. sham): 25.8% (8/31) vs. 17.6% (6/34) at 1 month (*p* = 0.424); 54.8% (17/31) vs. 35.1% (13/37) at 3 months (*p* = 0.142).	Median BCVA (logMAR; SRT vs. sham): baseline, 0.18 vs. 0.18; 1 month, 0.14 vs. 0.14 (*p* = 0.251); 3 months, 0.10 vs. 0.12 (*p* = 0.054).	3 months	No; retreatment permitted from month 3, after the primary endpoint assessment.	Yes; masked independent efficacy evaluators and OCT reading center.
Lee et al. (2021) [48]	Prospective randomized open-label controlled clinical trial	Chronic CSC; 22 SRT/22 untreated controls	Real-time feedback-controlled dosimetry using optoacoustic and reflectometric signals with automatic pulse termination; 527-nm Nd:YLF laser; 200-μm spot diameter; 1.7-μs pulse duration; up to 15 micropulses/spot (automatic termination); 100 Hz.	Mean preset pulse energy, 135.5 ± 22.2 μJ; mean delivered pulse energy, 95.5 ± 28.2 μJ.	6 weeks (before retreatment): Complete SRF resolution, 63.6% (14/22) vs. 23.8% (5/21) (SRT vs. control). 12 weeks (after retreatment/crossover): 70.3% (26/37) among all SRT-treated eyes.	6 weeks (SRT vs. control): BCVA, 0.23 ± 0.23 vs. 0.18 ± 0.14 logMAR; retinal sensitivity, 22.3 ± 4.0 vs. 20.0 ± 4.2 dB. Retinal sensitivity improved significantly from baseline in the SRT group (*p* = 0.002).	12 weeks	6 weeks: No. 12 weeks: Yes; repeat SRT and control-group crossover at week 6 for persistent SRF.	Not reported (open-label trial).
Büttner et al. (2021) [49]	Prospective observational study	Persistent acute CSC with symptoms lasting ≥6 months and persistent SRF despite prior eplerenone therapy; 17 eyes (16 patients)	Optoacoustic feedback-guided dosimetry with visible test-spot titration (treatment energy set at 80% of the first visible test-spot energy); 527-nm Nd:YLF laser; 200-μm spot diameter (in air); 1.7-μs pulse duration; 30 pulses/spot; 100 Hz.	Mean 132.35 ± 22.23 μJ (range, 90–170 μJ)	Complete SRF resolution: 35.3% (6/17) at 4 weeks; 58.8% (10/17) at 12 weeks; 64.7% (11/17) at final follow-up.	BCVA: 0.213 ± 0.196 logMAR at baseline to 0.12 ± 0.227 at 12 weeks (not significant); CRT decreased by 133.63 µm at 12 weeks (*p* = 0.001).	12 weeks	Yes; from week 4 for insufficient response. Three eyes retreated at week 4 before the 12-week outcome.	Not reported.
Yoon et al. (2024) [50]	Prospective real-world study	CSC; 52 eyes (47 chronic, 5 acute)	Real-time optoacoustic–reflectometric feedback-controlled dosimetry with automatic pulse termination; 527-nm Nd:YLF laser; 200-μm spot diameter; 1.7-μs pulse duration; up to 15 micropulses/spot; 100 Hz; pulse energy progressively increased within each burst.	Not reported.	Complete SRF resolution: 61.5% (32/52) at 6 months; 67.3% (35/52) at the last visit (up to 24 months).	Mean BCVA (logMAR): 0.15 ± 0.18 at baseline; 0.11 ± 0.17 at 3 months (*p* = 0.017); 0.12 ± 0.21 at 6 months (*p* = 0.062); 0.08 ± 0.23 at 24 months (*n* = 25; *p* = 0.041).	24 months	Yes; rescue PDT or anti-VEGF permitted for persistent/recurrent SRF (14/52 eyes received additional treatment); timing not reported.	Not reported.
Jeon et al. (2026) [51]	Prospective cohort study	Chronic CSC with symptoms lasting ≥3 months; 60 eyes enrolled, 52 eyes completing 3-month evaluation	Fundus image-based titration using delayed barely visible test spots on color fundus photography; 527-nm Nd:YLF laser; 200-μm spot diameter; 1.7-μs pulse duration; 10 micropulses/spot for titration and 3 micropulses/spot for treatment at the same pulse energy; 100 Hz.	Mean 139.62 ± 23.69 μJ (range, 80–200 μJ)	Complete SRF resolution at 3 months: 80.8% (42/52; complete-case analysis); 70.0% (42/60; all enrolled eyes, assuming missing eyes were nonresponders).	Mean BCVA (logMAR): 0.17 ± 0.15 at baseline; 0.09 ± 0.12 at 1 month; 0.08 ± 0.13 at 2 months; 0.06 ± 0.12 at 3 months (*p* < 0.001). Retinal sensitivity (MD): −0.89 ± 1.65 dB at baseline to 0.02 ± 1.29 dB at 3 months (*p* < 0.001).	3 months	Yes; at month 2 for persistent SRF (20/52 eyes, 38.5%), before the 3-month outcome.	Not reported.

Abbreviations: BCVA, best-corrected visual acuity; CRT, central retinal thickness; CSC, central serous chorioretinopathy; ETDRS, Early Treatment Diabetic Retinopathy Study; FA, fluorescein angiography; logMAR, logarithm of the minimum angle of resolution; MD, mean deviation; Nd:YLF, neodymium-doped yttrium lithium fluoride; OCT, optical coherence tomography; PDT, photodynamic therapy; SRF, subretinal fluid; SRT, selective retina therapy; VEGF, vascular endothelial growth factor. Note: Beam profiles were not reported in the included studies and are therefore not presented in the table. Büttner et al. used the term “persistent acute CSC” because the cases lacked characteristic atrophic changes in chronic CSC despite prolonged symptoms.

Because conventional FA-based titration requires delayed angiographic assessment and does not provide immediate feedback during irradiation, objective real-time dosimetry methods have been developed. Optoacoustic dosimetry was introduced to detect pressure transients generated during SRT irradiation and thereby provide real-time information on the RPE response [52]. Subsequently, feedback-controlled systems incorporated reflectometric detection with automatic energy ramping, progressively increasing pulse energy until an RPE response was detected, after which further irradiation was terminated [53]. These methods have been developed to facilitate real-time detection and individualized dosimetry of selective RPE injury and have been evaluated in experimental and clinical settings. However, their accuracy across different treatment conditions and their adoption in routine clinical practice remain subjects for further investigation [52,53,54].

More recently, a fundus image-guided SRT approach, using fundus image-based titration, has been introduced. This approach takes advantage of the observation that SRT lesions that are not clinically visible immediately after irradiation may develop delayed subtle changes that are detectable on color fundus photography. Test spots are delivered using different pulse energies and micropulse numbers, and delayed fundus images are then used to identify the lowest exposure producing a barely visible fundus change, with FA used to confirm the corresponding RPE response. In the reported protocol for CSC, the pulse energy producing a barely visible fundus change approximately 1 h after irradiation with 10 micropulses is subsequently used for treatment, with the number of micropulses reduced to three, thereby incorporating both pulse energy and micropulse number into individualized treatment selection [51,55]. Importantly, in both studies, test spots delivered with 3 micropulses at the selected laser setting exhibited FA-positive leakage, although less pronounced than that observed with 10 micropulses, providing angiographic evidence of RPE disruption despite the reduced micropulse number [51,55].

Experimental thermal modeling has demonstrated that both pulse number and repetition frequency influence heat accumulation during SRT, supporting the consideration of micropulse number along with pulse energy as important dosimetric parameters in SRT [56]. Because clinical studies of this fundus image-based titration approach have been conducted only in Asian populations to date, its applicability across populations with different fundus pigmentation remains to be established [51,55]. Furthermore, the consistency of FA-positive responses following exposure to 3 micropulses across different laser systems and independent clinical settings requires further validation.

### 4.4. Technical Differences Among RPE-Targeted Laser Modalities

A key methodological distinction among RPE-targeted laser modalities is the spatial distribution of treatment. SMPL protocols have employed treatment patterns ranging from relatively focal, leakage-directed application to denser treatment over broader affected areas, with substantial variation in treatment extent and distribution across protocols (Table 1). In reported clinical applications, NRT has similarly used a patterned, nondamaging treatment over the selected macular region, rather than targeting individual leakage sites [12,13,14].

**Figure 2:**
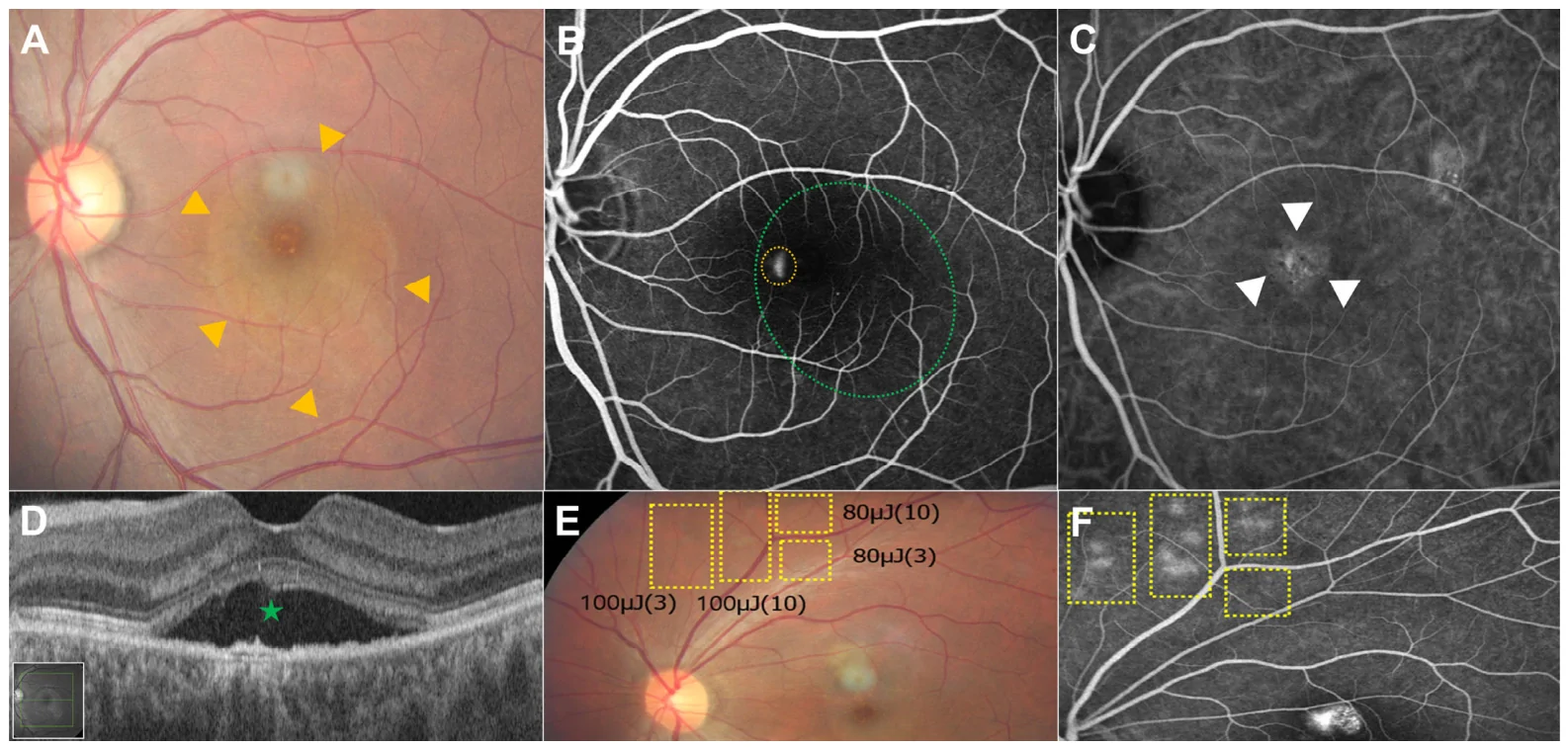
Spatial relationship between retinal pigment epithelium (RPE) leakage, subretinal fluid (SRF), and laser treatment areas, with an example of fundus image-based selective retina therapy (SRT) titration in central serous chorioretinopathy (CSC). (**A**–**D**) Clinical images from a representative case of CSC with focal leakage, with schematic overlays illustrating differences in treatment area. (**A**) Color fundus photograph showing the approximate extent of the serous retinal detachment involving the central macula (yellow arrowheads). (**B**) Late-phase fluorescein angiography (FA) showing focal leakage at the fovea. The yellow circle schematically represents a leakage-directed treatment area for SRT, whereas the green circle represents a broader treatment area that may be used for subthreshold micropulse laser or nondamaging retinal laser therapy. These overlays are intended to illustrate potential differences in treatment distribution and do not represent the exact treatment performed in this eye, individual laser spot size, or precise treatment boundaries. (**C**) Late-phase indocyanine green angiography demonstrating choroidal hyperfluorescence (white arrowheads) extending over a broader area than the focal fluorescein leakage, illustrating the spatial difference between choroidal abnormalities and focal RPE leakage in this representative case. (**D**) Optical coherence tomography demonstrating fovea-involving SRF (green star). (**E**,**F**) Images from a separate case illustrating fundus image-guided titration, in which test spots were placed near the vascular arcade to determine the appropriate laser energy before therapeutic SRT was applied to macular leakage sites, as previously described [51,55,57]. (**E**) Color fundus photography obtained 1 h after test spot irradiation shows multiple test spot pairs within the yellow rectangular boxes. Test spots delivered at 100 μJ with 10 micropulses were barely visible, whereas some corresponding spots delivered at 100 μJ with 3 micropulses were invisible on color fundus photography. (**F**) Corresponding FA obtained 1 h after irradiation demonstrates less pronounced leakage from test spots delivered at 100 μJ with 3 micropulses than from those delivered at the same pulse energy with 10 micropulses. Based on the titration protocol, 100 μJ was selected as the lowest pulse energy producing delayed, barely visible test spots with 10 micropulses, and the micropulse number was reduced from 10 to 3 at the same pulse energy for therapeutic SRT. Panels E and F were adapted from Kim et al. [57], under the terms of the Creative Commons Attribution 4.0 International License (CC BY 4.0).

In contrast, SRT is primarily FA-leakage-directed, with treatment spots applied to angiographically identified leakage sites or areas, typically with separation between adjacent spots (Table 2). Consequently, in cases of focal CSC, this strategy may involve a smaller treated area than broader SMPL or NRT treatment, whereas this distinction may be less pronounced in eyes with multifocal or diffuse leakage. The spatial relationship between RPE leakage, SRF, and SRT treatment, together with an example of fundus image-based SRT titration, is illustrated in Figure 2.

Foveal involvement also depends on the treatment pattern and location of the targeted abnormality. Sublethal approaches, such as SMPL and NRT, may permit treatment within the central macular region because treatment is intended to remain below the threshold resulting in visible retinal injury. In SRT, treatment distribution is primarily determined by the location of angiographically identified leakage. The principal technical characteristics of these modalities are summarized in Table 3.

## 5. Clinical Evidence for RPE-Targeted Laser Therapy in CSC

Clinical outcomes reported for RPE-targeted laser therapy should be interpreted cautiously because of the substantial differences across studies. Direct comparison of complete SRF resolution rates among SMPL, NRT, and SRT is limited by differences in study design, inclusion and exclusion criteria, disease chronicity, baseline SRF characteristics, treatment protocols, retreatment criteria, and timing of outcome assessment.

In particular, studies including acute CSC may be influenced by spontaneous SRF resolution, whereas those involving chronic CSC cohorts may differ considerably in terms of disease duration and prior treatment. Complete SRF resolution is therefore presented as an important anatomical outcome within individual studies, rather than as a basis for direct efficacy comparison among laser modalities.

### 5.1. SMPL

SMPL has been evaluated in several controlled clinical studies of CSC, including randomized controlled trials, using different wavelengths, dosimetry strategies, treatment patterns, and comparators (Table 1).

Among comparative studies, the multicenter PLACE trial randomized 179 patients with chronic CSC to high-density SMPL or half-dose photodynamic therapy (PDT). Complete SRF resolution was significantly less frequent after SMPL than after half-dose PDT, occurring in 13.8% versus 51.2% of patients at 6–8 weeks and in 28.8% versus 67.2% at the final evaluation at 7–8 months [20]. Notably, the PLACE trial maintained a 500-μm distance from the foveal center during SMPL treatment, whereas PDT could include the fovea. The investigators acknowledged that this difference might have influenced the study outcomes, although they considered its impact likely to be minimal because the excluded foveal region represented only a small portion of the relatively extensive indocyanine green angiography-guided treatment area [20]. In a subsequent follow-up study restricted to patients who had achieved complete SRF resolution in the PLACE trial, van Rijssen et al. found no significant between-group differences in BCVA, retinal sensitivity, or vision-related quality of life at approximately 20 months after treatment, despite a higher rate of sustained SRF resolution after half-dose PDT than after SMPL (93% vs. 53%; *p* = 0.006) [58].

Brelen et al. conducted a prospective, double-masked randomized controlled trial comparing 577-nm SMPL with half-dose PDT in chronic CSC. Complete SRF resolution occurred in 24.2% versus 58.8% of patients at 1 month and 82.1% versus 90.9% at 12 months, respectively. Although the difference in complete SRF resolution was not significant at 12 months, half-dose PDT achieved significantly faster SRF resolution (*p* = 0.016) [42]. In the study by Kretz et al., 810-nm micropulse treatment reduced fluorescein leakage activity in 60.0% of treated eyes at 16 weeks, as compared with 66.7% after half-dose PDT, with BCVA improvements of 6.7 and 8.5 ETDRS letters, respectively [37]. Sun et al. compared 577-nm SMPL with conventional laser; they reported complete SRF resolution in 63.6% and 81.8% of eyes, respectively, at 12 weeks, with comparable visual improvement [39]. In an earlier retrospective comparative study of 100 eyes with chronic CSC, Scholz et al. reported a greater reduction in central retinal thickness after 577-nm SMPL than after half-dose PDT at 6 weeks (148 vs. 76 μm; *p* = 0.041), whereas the difference in complete SRF resolution (36% vs. 21%) was not statistically significant (*p* = 0.095) [59]. Overall, these studies demonstrate anatomical improvement following SMPL, although comparative trials with PDT have generally shown greater or more rapid SRF resolution with PDT, particularly at earlier follow-up.

Randomized studies specifically involving acute CSC should be interpreted separately, because spontaneous SRF resolution is common. Zhou et al. found broadly comparable visual and anatomical outcomes between 577-nm SMPL and conventional laser [40]. In a subsequent randomized non-inferiority trial comparing 577-nm SMPL with half-dose PDT in cases of acute CSC, complete SRF resolution by 6 months occurred in 82.4% and 91.2% of eyes, respectively, while SMPL met the prespecified non-inferiority criterion for visual acuity improvement [41]. Additionally, Arora et al. compared 810-nm SMPL directly with observation alone in acute CSC and reported a lower rate of recurrent or persistent neurosensory detachment by 6 months (11.8% vs. 29.4%; *p* = 0.036), together with significantly better visual acuity, in the SMPL group [38].

Retreatment has been incorporated into SMPL protocols for persistent SRF, although the timing and criteria for further treatment have varied across studies. Treatment was repeated for persistent macular SRF at 6–8 weeks in the PLACE trial and at 3 months in the study by Zhou et al. [20,40]. In the randomized trial by Brelen et al., repeat SMPL was considered at months 3 and 6 for persistent SRF, with additional treatment permitted at month 9 according to the study protocol [42]. Although repeated SMPL has been incorporated into clinical study protocols, evidence regarding the long-term effects of repeated treatment remains limited.

### 5.2. NRT

Clinical evidence for NRT in CSC remains limited. Lavinsky and Palanker initially evaluated 577-nm continuous-wave laser treatment using EpM in 16 eyes with persistent CSC (symptom duration >4 months), applying subvisible laser energy in a high-density macular pattern. Complete SRF resolution was reported in 75% of eyes by 6 months, without clinically or multimodally detectable retinal damage [14].

Subsequent prospective studies have further evaluated anatomical and functional outcomes with EpM-based NRT. Schworm et al. evaluated a standardized macular treatment pattern in 42 eyes with chronic CSC and reported achieving complete SRF resolution in 42.9% of eyes at 6 months and in 53.8% at 12 months, accompanied by significant improvements in BCVA and retinal sensitivity [60]. Kaderli et al. evaluated 14 treated eyes and 18 untreated controls and reported complete SRF resolution in 71.4% of treated eyes at 3 months, with significant improvements in BCVA and anatomical outcomes [61]. Lee et al. subsequently evaluated a more localized treatment protocol in 31 Korean eyes with chronic CSC and reported achieving complete SRF resolution in 48.4% of eyes at 6 months, together with significant reductions in central macular thickness and SRF height [62].

Retreatment has also been used for persistent or recurrent SRF following NRT. In the study by Schworm et al., retreatment was performed at 3-month intervals when SRF persisted [60]. Lee et al. similarly reported further reduction in SRF height after retreatment of eyes with persistent SRF [62]. In a more recent prospective study of 11 eyes, complete SRF resolution after a single treatment was achieved in 4 eyes (36.4%), whereas 7 eyes (63.6%) required additional treatment for persistent SRF; SRF resolution was reported in all eyes after the final treatment [63].

Overall, the available clinical evidence for NRT in CSC is based on relatively small studies that have been predominantly nonrandomized. Randomized comparative trials specifically evaluating NRT in CSC remain lacking.

### 5.3. SRT

Early retrospective studies provided initial clinical evidence for SRT in CSC. Elsner et al. evaluated 27 eyes with active CSC and reported complete SRF resolution in 85.2% at 4 weeks following SRT [64]. Subsequently, Kim et al. evaluated 16 eyes with acute CSC using real-time feedback-controlled dosimetry and reported complete SRF resolution in all eyes at 3 months [65]. However, these uncontrolled findings should be interpreted cautiously, particularly given the possibility of spontaneous resolution in acute CSC.

SRT has been evaluated in randomized controlled trials and in several prospective clinical studies using different dosimetry and titration approaches with individualized treatment pulse energies in both acute and chronic CSC (Table 2). In a randomized trial involving patients with CSC symptoms persisting for at least 3 months, Klatt et al. reported significantly greater BCVA improvement following SRT than observation (12.7 vs. 6.3 ETDRS letters; *p* = 0.04), along with a significantly greater reduction in SRF thickness (203 vs. 41 μm; *p* = 0.005). Complete SRF resolution at 3 months was observed in 71.4% of SRT-treated eyes compared with 40.0% of untreated controls, although the between-group difference was not statistically significant (*p* = 0.081) [45].

In a multicenter, randomized, sham-controlled trial, Oh et al. reported complete SRF resolution at 3 months in 54.8% of SRT-treated eyes and 35.1% of sham-treated eyes, without a statistically significant between-group difference (*p* = 0.142). Nevertheless, SRT was associated with significantly greater early reductions in SRF height and central macular thickness at 1 month (*p* = 0.0029 and *p* = 0.0007, respectively). Thus, although the primary endpoint of complete SRF resolution was not met, the study demonstrated favorable early anatomical outcomes [47].

In a randomized, open-label controlled trial involving 44 patients with chronic CSC, Lee et al. demonstrated a significantly higher rate of complete SRF resolution at 6 weeks in the SRT group than in the observation group (63.6% vs. 23.8%, *p* = 0.020). Mean SRF height at 6 weeks was also significantly lower in the SRT group (*p* = 0.041), whereas the between-group difference in BCVA was not statistically significant. These findings support a short-term anatomical benefit of SRT, although the open-label design, modest sample size, and limited follow-up should be considered when interpreting the results [48]. In a prospective real-world study, Yoon et al. reported complete SRF resolution in 62% of eyes at 6 months following SRT, with real-time feedback-controlled dosimetry, with follow-up extending to 24 months [50].

Fundus image-guided SRT was subsequently evaluated prospectively in 60 eyes with chronic CSC. Among the 52 eyes completing the 3-month evaluation, complete SRF resolution was observed in 42 eyes (80.8%) following a treatment protocol that permitted additional SRT at 2 months for persistent SRF. Significant improvements in BCVA, central foveal thickness, SRF height, and retinal sensitivity were also reported. However, the single-arm design, incomplete follow-up, and allowance for retreatment limit the interpretation of these outcomes as evidence of treatment efficacy [51].

Retreatment has also been used for persistent SRF following SRT. In an earlier prospective study, Framme et al. performed repeat SRT in six eyes with chronic-recurrent CSC and persistent disease after initial treatment, with subsequent SRF resolution reported in five of the six retreated eyes [46]. In a prospective study, 20 of 52 eyes (38.4%) underwent retreatment at 2 months [51]. These findings indicate that retreatment may be necessary in a substantial proportion of eyes with persistent SRF, although retreatment criteria and timing varied across studies.

Definitions of CSC chronicity varied considerably across the clinical studies reviewed above. Arora et al. [38] defined acute CSC as symptom duration of less than 2 months, whereas Zhou et al. [40] used a threshold of less than 6 months. In contrast, Klatt et al. [45] classified CSC with symptoms persisting for at least 3 months as acute, and Büttner et al. [49] used the term “persistent acute CSC” for cases lasting at least 6 months. Brelen et al. [42] and Jeon et al. [51] classified CSC lasting more than 3 months as chronic. These discrepancies illustrate the difficulty of applying a uniform acute–chronic classification retrospectively. Consequently, study populations may overlap despite different diagnostic labels, and comparisons of treatment outcomes should be interpreted cautiously, particularly given the influence of disease duration and spontaneous SRF resolution. Future studies should clearly specify their chronicity criteria rather than relying solely on the terms acute or chronic.

Overall, although some randomized studies have demonstrated favorable anatomical outcomes following SRT, its superiority over observation has not been consistently established, and direct randomized comparisons with PDT remain unavailable.

### 5.4. Safety of RPE-Targeted Laser Therapy

A major rationale for RPE-targeted laser therapy is to minimize collateral damage to the neurosensory retina while producing a therapeutic response at the RPE level. Across clinical studies of CSC using SMPL, NRT, or SRT, serious treatment-related ocular adverse events have been uncommon, although the extent and methods of safety assessment have varied among modalities.

For SMPL, clinical studies have generally reported no clinically significant laser-induced retinal damage. Functional safety has also been assessed using microperimetry and other functional measures. Yadav et al. reported no evidence of RPE or retinal damage on optical coherence tomography (OCT), FA, or fundus autofluorescence (FAF), with preservation or improvement of retinal sensitivity on microperimetry [66]. A subsequent prospective study of trans-foveal SMPL assessed microperimetry, contrast sensitivity, and multifocal electroretinography and found no morphological or functional adverse effects following treatment [67]. In a larger clinical series of 101 eyes with CSC, Chhablani et al. evaluated multiple parameter sets of navigated microsecond pulsing laser and reported no laser-induced retinal damage or treatment-related adverse events during follow-up, including in eyes undergoing retreatment [68]. However, RPE hyperplasia with associated visual loss has also been reported following SMPL, with higher delivered fluence considered a possible contributing factor [69]. Paradoxical worsening of central retinal edema following SMPL has also been described in a case report by Gawęcki et al. [70]. Nevertheless, overall, clinical studies have generally reported favorable safety outcomes for SMPL, although treatment parameters, dosimetry, and methods of safety assessment have varied among studies.

Available clinical studies using NRT have generally reported favorable safety outcomes. In the initial clinical study of EpM-guided nondamaging continuous-wave laser therapy for chronic CSC, no clinically apparent or multimodally detectable retinal damage attributable to treatment was observed [14]. Subsequent studies have reported similar favorable safety profiles [60,62]. In a recent prospective study, pigmentary RPE changes were observed in 5 of 11 eyes (45.4%), although their relationship to laser treatment versus the natural course of CSC was uncertain. No RPE atrophy, choroidal neovascularization, or other retinal or choroidal complications were reported [63]. Overall, available safety evidence for NRT remains based on relatively small cohorts.

For SRT, selective RPE injury is intentional, making preservation of the overlying neurosensory retina an important safety consideration. Prospective clinical studies using different dosimetry approaches in SRT have reported no serious treatment-related ocular adverse events [45,47,48,51]. Functional safety has also been evaluated using microperimetry. Localized pigmentary changes may nevertheless be observed at SRT test spots, consistent with an RPE-level tissue response. Such changes may evolve on color fundus photography and FAF during follow-up, without corresponding scotomatous changes on microperimetry [48,51,55]. Consistently, microperimetric assessment of test spots placed near the vascular arcade, outside the diseased macular region, has demonstrated no persistent reduction in retinal sensitivity [71]. These findings support preservation of localized neurosensory retinal function following selective RPE injury.

In several clinical studies of SRT for CSC, treatment was directed toward angiographically identified leakage sites without specifying a fixed minimum distance from the foveal center or a predefined foveal exclusion zone [45,47,48,51,55]. These studies did not report clinically significant retinal damage or functional deterioration attributable to SRT. Treatment near or within the central fovea requires careful selection of treatment parameters, and the safety of direct foveal irradiation has not been systematically evaluated.

In the randomized study by Lee et al. [48], retinal sensitivity was measured using MAIA microperimetry (CenterVue, Padova, Italy), with two baseline examinations performed to minimize learning effects. No significant change in retinal sensitivity was observed in the untreated control group. In contrast, Jeon et al. [51] used COMPASS microperimetry (CenterVue, Padova, Italy), which automatically calculates mean deviation (MD). The relatively preserved baseline MD (−0.89 ± 1.65 dB), despite persistent SRF, should be interpreted in the context of the study population and the use of an averaged functional index. However, a learning effect cannot be excluded when interpreting the improvement in MD in this single-arm study.

While some longer-term clinical outcomes, including those after retreatment, have been reported [72,73], the evidence regarding the long-term safety of repeated SRT remains limited.

## 6. Mechanistic Implications of RPE-Targeted Laser Therapies in CSC

The biological responses induced by RPE-targeted laser therapies differ according to their intended tissue effects. SMPL and NRT are primarily designed to induce sublethal cellular modulation, whereas SRT is intended to produce selective RPE injury followed by repair and repopulation. However, these categories represent intended biological endpoints rather than completely exclusive tissue responses, and the actual effects may vary with laser parameters and delivered energy.

### 6.1. Sublethal RPE Modulation and Cellular Stress Responses

SMPL is intended to induce biological responses in the RPE through controlled thermal stimulation without overt photocoagulative injury. Experimental studies have demonstrated changes in cellular stress responses and gene expression following SMPL irradiation. Shiraya et al. reported heat shock protein expression and distinct transcriptomic responses in cultured human RPE cells following SMPL, suggesting that sublethal thermal stimulation can activate molecular pathways without requiring extensive cellular injury [31]. In a mouse model, Hirabayashi et al. observed increased aquaporin-3 expression after SMPL irradiation without detectable retinal damage, suggesting a potential relationship between sublethal laser stimulation and fluid transport mechanisms [30].

However, the distinction between sublethal modulation and tissue injury is not absolute. Pollack et al. demonstrated variable RPE and outer retinal alterations following clinically undetectable micropulse laser irradiation [74]. Similarly, Yu et al. reported histological retinal changes after subthreshold micropulse irradiation at both 532 and 810 nm [75]. These findings indicate that the biological effects of micropulse irradiation depend on treatment parameters and delivered dose, and that the absence of visible lesions does not necessarily exclude microscopic tissue injury. Accordingly, tissue responses to SMPL and SRT may overlap under certain irradiation conditions. However, the available evidence does not establish RPE injury or subsequent repopulation as a therapeutic mechanism of SMPL. Although experimental findings support the biological plausibility of sublethal RPE modulation, its direct contribution to restoration of RPE barrier function and SRF resolution in human CSC remains to be established.

NRT similarly aims to induce sublethal RPE responses through controlled photothermal stimulation, but its treatment parameters are determined using a computational model of retinal thermal damage rather than direct confirmation of the biological response at individual treatment sites [12,13]. Although this model-based approach provides a systematic framework for dosimetry, the predicted treatment endpoint does not directly establish the absence of microscopic tissue injury or the activation of specific RPE stress-response pathways. Furthermore, the relationship between model-predicted thermal exposure, actual RPE responses, and SRF resolution has not been directly established in human CSC. Thus, as with SMPL, the proposed therapeutic mechanisms of NRT should be distinguished from its observed clinical outcomes.

### 6.2. RPE Repair and Fluid Homeostasis After SRT

Unlike the primarily sublethal modulation intended with SMPL and NRT, SRT is designed to induce selective RPE injury followed by a reparative response, which has been investigated in experimental studies.

Experimental studies have demonstrated that the selectively injured RPE undergoes active repair after SRT through migration and proliferation of surrounding RPE cells, followed by repopulation of the treated area. Histological and ultrastructural studies have shown progressive restoration of RPE coverage and cellular architecture, while the overlying neurosensory retina remains largely preserved. RPE cell migration and proliferation have been observed to occur within several days after SRT, followed by restoration of the RPE monolayer and microvilli within approximately 1 week and subsequent recovery of ultrastructural features, including the tight junctions [17,54,76]. These findings form the biological basis for the concept of RPE repair and regeneration following SRT.

Clinical imaging provides complementary, indirect evidence of this reparative process. Early autofluorescence studies have demonstrated that ophthalmoscopically invisible SRT lesions initially appear hypoautofluorescent and subsequently become hyperautofluorescent during follow-up [77]. Long-term multimodal imaging has similarly demonstrated decreased FAF shortly after SRT, followed by increased FAF at approximately 1 month, and gradual fading of autofluorescence thereafter. OCT changes involving the RPE and interdigitation zone gradually regressed during long-term follow-up, without RPE atrophy [78]. A similar temporal FAF pattern, with no change or with hypoautofluorescence shortly after irradiation followed by increased autofluorescence at 1 month and subsequent attenuation, has also been observed with fundus image-guided SRT [55]. These sequential imaging changes are consistent with progressive RPE remodeling occurring after selective injury, although they do not directly demonstrate cellular regeneration.

Post-SRT RPE repair may also involve biological responses beyond cellular repopulation. Experimental studies have demonstrated the release of mediators, including matrix metalloproteinase-2 and pigment epithelium-derived factor, during post-SRT RPE regeneration, suggesting that extracellular matrix remodeling and paracrine signaling may accompany the reparative process [79,80]. The extent to which these responses contribute to restoration of the RPE barrier or fluid transport function in CSC, however, remains uncertain.

In CSC, this reparative response may have functional consequences beyond structural restoration of the treated RPE. Repopulation of the selectively injured RPE may contribute to restoration of local barrier integrity after SRT [17,54,76]. Restoration of RPE integrity may, in turn, contribute to normalization of fluid transport across the RPE, facilitating SRF resolution, consistent with the established role of the RPE in SRF homeostasis and the proposed alterations in RPE fluid transport in CSC [18,23,24,25].

This reparative response involves migration and proliferation of neighboring RPE cells following selective injury [17]. However, direct evidence that RPE repopulation restores physiological function in human CSC remains limited. Thus, this functional effect should be regarded as biologically plausible rather than definitively established.

### 6.3. Leakage-Directed Treatment and Resolution of SRF

A distinctive feature of SRT for CSC is that treatment can be directed primarily toward angiographically identified RPE leakage rather than to the entire area of neurosensory detachment. In eyes with focal leakage, the treated RPE area may therefore be substantially smaller than the area occupied by SRF. SRF resolution has been reported following localized leakage-directed SRT, although these clinical observations do not establish whether the response results specifically from restoration of RPE barrier function at the treated leakage sites [45,46,47,48,51].

The spatial relationship between leakage and treatment becomes more complex in eyes with multifocal or diffuse leakage, where a larger RPE area is involved and a greater number of treatment spots may be required. Kyo et al. reported that focal leakage was independently associated with a greater likelihood of complete SRF resolution after SRT [81]. However, the treatment response appears to depend on factors beyond leakage pattern alone. In a larger chronic CSC cohort, Kim et al. found that a greater baseline SRF height independently predicted the need for retreatment, whereas the leakage type did not [82]. These findings suggest that the clinical response to SRT may reflect both the spatial characteristics of the RPE leakage and the baseline burden of SRF.

The spatial disparity between the treated RPE area and the extent of SRF may also have mechanistic implications. Experimental studies have shown that selective RPE injury is followed by rapid migration and remodeling of the surrounding viable RPE cells. In the original histological study by Roider et al., RPE cells adjacent to the lesion extended toward the irradiated area within hours after selective injury, and the treated lesions became progressively covered by remodeled RPE cells during the subsequent healing process [15]. More recently, longitudinal multimodal imaging of human SRT lesions demonstrated findings that were considered to be consistent with closure of the laser-induced RPE defect through hypertrophy and migration of neighboring RPE cells [78]. These experimental findings and clinical imaging observations are consistent with RPE remodeling after SRT, although imaging findings alone cannot confirm restoration of normal RPE cellular structure or function.

In CSC, because SRT lesions are deliberately created at angiographically identified leakage sites, this reparative response may also contribute to restoration of local outer blood–retinal barrier integrity at the treated leakage site. Kim et al. proposed that the RPE layer that is newly formed after SRT may improve outer blood–retinal barrier integrity and that closure of leaking points by repopulated RPE cells may contribute to SRF resolution [82]. Sealing of the RPE leakage point has also been proposed as a therapeutic objective of leakage-directed SRT in CSC [49]. This concept is consistent with the proposed restoration of the RPE barrier following SRT targeting the dysfunctional RPE at leakage sites [51]. The physiological link between such localized RPE repair and SRF resolution, however, remains incompletely understood.

This spatially focal response may differ conceptually from the broader RPE functional modulation that has been proposed for SMPL and NRT. From a fluid-homeostatic perspective, restoration of barrier integrity at the treated leakage site could reduce abnormal inward fluid entry into the subretinal space, potentially allowing residual SRF to be cleared through fluid transport by the surrounding functional RPE. A potentially complementary hypothesis is “pump reversal” of RPE fluid transport, although this phenomenon has not been directly demonstrated in human CSC. Under normal conditions, the RPE actively transports fluid from the subretinal space toward the choroid [23,24,83], whereas this transport direction has been hypothesized to reverse in CSC, potentially in association with increased choroidal hydrostatic pressure and resistance to outward fluid transport [18,23,28].

One proposed hypothesis is that selective RPE injury followed by repopulation after SRT may influence local barrier integrity and fluid transport at treated leakage sites [18,23,49,51,55,82]. However, neither abnormal inward fluid transport at these sites nor its normalization following SRT has been directly demonstrated in human CSC. Therefore, the contribution of RPE repopulation and associated barrier changes to SRF resolution remains hypothetical, particularly in the presence of persistent underlying choroidal abnormalities.

### 6.4. PED and Choroidal Structural Responses

The high rates of SRF resolution following PDT may be explained by the balance between choroidal fluid entry and RPE-mediated fluid clearance, as proposed in the recently described “Tap–Drain” model of CSC [28]. In this conceptual framework, SRF accumulates when increased fluid entry associated with choroidal hyperpermeability and hemodynamic abnormalities exceeds the residual fluid clearance capacity of the RPE. By reducing choroidal hyperpermeability, PDT may decrease fluid entry sufficiently to allow the remaining RPE transport function to promote SRF resorption, without requiring direct induction of RPE injury or repopulation. This mechanism may help explain the favorable anatomical outcomes of PDT despite its predominantly choroidal therapeutic target. However, the relative contributions of reduced choroidal fluid entry and preserved RPE fluid clearance to SRF resolution after PDT have not been directly established, and this interpretation remains a pathophysiological hypothesis.

Beyond the mechanisms governing SRF accumulation and resolution, changes in specific RPE structural abnormalities provide an additional perspective on the interaction between choroidal dysfunction and RPE integrity. PED represents an anatomical separation of the RPE from the underlying Bruch’s membrane and is frequently observed in CSC, where its development has been associated with underlying choroidal abnormalities [3,84]. Persistent PED has been associated with worse long-term visual outcomes [85], while the natural history of CSC-associated PED further suggests that anatomical resolution does not necessarily indicate restoration of RPE integrity. Mudvari et al. [86] reported that, despite spontaneous resolution of PED in 65% of cases during a mean follow-up of 49 months, localized RPE atrophy developed in 86% of these resolved cases, and subfoveal PEDs were associated with a worse visual prognosis. PED morphology may therefore serve as an additional structural marker for examining the interaction between choroidal dysfunction and RPE-level responses to treatment.

In CSC, PED may respond to treatments that have different primary tissue targets. In a secondary analysis of the PLACE trial [87], both half-dose PDT and high-density SMPL were associated with reductions in PED height, although the reduction was significantly greater with half-dose PDT. With these treatments, complete resolution of the highest macular PED at 7–8 months occurred in 36.5% and 13.0% of eyes, respectively. A separate retrospective study of 577-nm SMPL also demonstrated a significant reduction in the OCT-measured PED area at 6 months, providing additional evidence that PED morphology may change following SMPL treatment [88]. The mechanisms underlying these changes may differ according to the primary tissue target, with PDT acting primarily at the choroidal level and SMPL primarily modulating RPE function. Nevertheless, the specific mechanisms underlying PED reduction remain uncertain.

In an early case series of five eyes with CSC-associated serous PED, Klatt et al. reported complete SRF resolution in four eyes following SRT directed toward PED-associated leakage, despite persistence of the PED itself [89]. More recently, a retrospective study from our group reported that SRT directed toward PED-associated leakage was associated with significant reductions in both PED height and SRF, without a significant change in subfoveal choroidal thickness [57]. In this retrospective series of 32 eyes, complete SRF resolution was observed in 78.1% of eyes at 3 months, and reductions in PED and SRF heights were moderately correlated. These findings suggest an association between PED remodeling and restoration of SRF homeostasis, although they do not establish a causal relationship. Given the above-mentioned post-SRT RPE reparative response, the observed PED changes are also consistent with a potential contribution from localized RPE remodeling. Because PED directly reflects displacement of the RPE from Bruch’s membrane, this possibility is biologically plausible, but has not been directly demonstrated in vivo.

These observations should not be interpreted as direct comparisons among treatment modalities given the differences in study design, patient selection, outcome measures, and follow-up. Moreover, CSC-associated PED may spontaneously reduce or resolve, and PED improvement observed in uncontrolled or single-arm studies cannot be reliably distinguished from the natural course of the disease. The PED responses reported after RPE-targeted laser treatment should be considered supportive observations that warrant further investigation. Prospective controlled studies using standardized PED measurements are needed to determine the treatment-attributable effects of RPE-targeted interventions and to clarify whether PED remodeling is associated with SRF resolution and long-term preservation of RPE integrity.

Clinical studies using SRT have demonstrated SRF improvement in the absence of significant subfoveal choroidal thickness changes [48,51,90], suggesting that fluid resolution following SRT can occur without measurable changes in choroidal thickness. However, another clinical series reported significant reduction in central choroidal thickness after SRT [91], indicating that choroidal thickness responses after SRT may not be uniform across studies.

Studies of SMPL have likewise shown variable choroidal responses. Significant reductions in choroidal thickness have been reported in some studies [39,92], whereas others found no significant change in subfoveal choroidal thickness, despite anatomical improvement [93,94]. In an OCTA analysis of 33 patients from the randomized trial subsequently reported in full by Brelen et al. [42], Ho et al. [43] observed reductions in choriocapillaris flow deficit areas following both SMPL and half-dose PDT. These reductions occurred earlier after PDT than after SMPL, while a significant reduction in foveal choroidal volume was observed only in the PDT group.

To our knowledge, longitudinal OCTA studies evaluating choriocapillaris changes after SRT for CSC have not yet been reported. Therefore, whether SRF resolution following SRT is accompanied by changes in choriocapillaris perfusion remains unclear.

Overall, these findings indicate that RPE-targeted laser therapies may be accompanied by changes in PED morphology as well as variable choroidal structural responses. The relationship of these structural changes to SRF resolution and their underlying mechanisms remains incompletely understood. Notably, improvement or resolution of SRF was not consistently accompanied by a measurable reduction in choroidal thickness across these studies.

## 7. Future Directions in RPE-Targeted Laser Therapy

Future development of RPE-targeted laser therapy for CSC will require optimization of the biological response produced by each laser modality. Although SMPL, NRT, and SRT share the RPE as the principal therapeutic target, they induce different degrees and patterns of RPE response, ranging from sublethal cellular modulation to selective RPE injury followed by repair. Optimization of these treatments should focus not only on laser parameters, but also on defining the appropriate tissue response for each modality and determining how that response relates to clinical efficacy.

Treatment protocols should be refined to achieve a reproducible therapeutic RPE response, while minimizing unintended retinal injury. This issue is particularly relevant because treatment parameters, dosimetry, and methods for determining the therapeutic endpoint have not been fully standardized across RPE-targeted laser modalities. In addition to exposure parameters, the treatment pattern and spatial coverage may influence the biological response elicited in the RPE and should therefore be standardized and reported in future studies. The recently proposed Regenerative Retinal Laser and Light Therapies framework provides a systematic approach for describing these treatments according to treatment pattern, dosimetry, exposure parameters, and the underlying laser–tissue interaction, which may improve reproducibility and comparability across future studies [95].

Identifying the patients who are most likely to respond to RPE-targeted treatment is also important. To date, studies have evaluated clinical and imaging features, such as leakage pattern, chronicity, PED, baseline SRF characteristics, and the extent of pre-existing RPE alteration. In an early prospective SMPL series, treatment response differed according to the angiographic RPE phenotype, with more favorable outcomes noted for eyes with point-source leakage without associated RPE atrophy than for those with RPE atrophy or with diffuse RPE decompensation [96]. A post hoc subgroup analysis of the PLACE trial also showed numerically higher complete SRF resolution after SMPL in eyes with focal than in those with diffuse leakage (38% vs. 21% at the final visit), raising the possibility that the leakage phenotype may contribute to the heterogeneity in response to SMPL, although this association requires further validation [97]. However, no single clinical or imaging phenotype has consistently predicted treatment response across RPE-targeted modalities.

Multimodal imaging-based CSC classification increasingly emphasizes the extent of RPE alteration, outer retinal integrity, and foveal involvement. This suggests that the RPE phenotype may provide a useful basis for future patient selection and stratification for RPE-targeted therapy [98].

Available long-term studies provide some evidence regarding treatment durability in CSC. In the long-term follow-up of the PLACE trial, SRF recurrence occurred in 7% of patients treated with half-dose PDT compared with 47% treated with high-density SMPL at approximately 20 months after treatment [58]. For SRT, Yoon et al. [50] reported that 27% of eyes received additional treatments, including PDT or anti-VEGF injections, during a 24-month follow-up. Lindziute et al. [73] reported that 50% of eyes required SRT retreatment, with retreatment-free survival rates of 57% at 1 year and 49% at 3 years. However, differences in study design, retreatment criteria, and follow-up duration limit direct comparisons of treatment durability across modalities.

Despite these findings, further long-term evaluation remains important, because most studies to date have emphasized relatively short-term anatomical outcomes. The durability of SRF resolution, recurrence, retreatment, retinal sensitivity, photoreceptor integrity, and the safety of repeated RPE intervention should be evaluated to determine whether anatomical improvement translates into sustained functional benefit.

Furthermore, the clinical evidence base requires strengthening. Many studies of RPE-targeted laser therapy have been relatively small, single-center, or heterogeneous in terms of treatment protocols, and adequately powered randomized trials using standardized treatment and outcome criteria remain limited. Future trials should evaluate the efficacy and safety of individual RPE-targeted modalities, using well-defined treatment protocols and clinically meaningful anatomical and functional outcomes. Comparative studies using established choroid-directed treatments may also help to define the clinical settings and patient phenotypes in which RPE-targeted treatment may play a complementary therapeutic role.

## 8. Conclusions

The contemporary understanding of CSC emphasizes the central role of choroidal vascular and hemodynamic abnormalities. Choroid-directed treatment, particularly PDT, remains the preferred therapeutic approach for chronic disease. Nevertheless, the accumulation and resolution of SRF ultimately depend on fluid homeostasis across the RPE, providing a biological rationale for therapeutic intervention at the RPE level.

SMPL, NRT, and SRT target the RPE through distinct laser–tissue interactions and biological responses, ranging from sublethal cellular modulation to selective RPE injury followed by repair and repopulation. Available evidence supports the biological plausibility that modulation or restoration of RPE barrier and fluid transport functions may contribute to SRF resolution. RPE-targeted laser treatments may therefore exert therapeutic effects in CSC by targeting RPE-level abnormalities and leakage as clinically relevant components of fluid dysregulation.

Nevertheless, their distinct biological mechanisms should not be interpreted as evidence of established clinical superiority. A 2025 Cochrane network meta-analysis found no clear evidence of superiority among the CSC interventions evaluated [22], while a separate assessment of systematic reviews characterized the evidence supporting SRT for chronic CSC as limited and inconclusive [99]. Although individual SRT trials have reported favorable anatomical outcomes, the comparative clinical efficacy of SRT remains to be established through adequately powered, masked randomized controlled trials. The relationship between choroidal abnormalities and RPE dysfunction, the biological response required for clinical efficacy, and the clinical and imaging phenotypes associated with treatment response remain incompletely defined. Future studies should clarify these relationships, optimize treatment parameters and treatment strategies, and determine the long-term efficacy and safety of repeated RPE-targeted interventions.

## Figures and Tables

**Table 3:** Technical characteristics of retinal pigment epithelium-targeted laser modalities used in central serous chorioretinopathy.

Characteristic	SMPL	NRT	SRT
Laser delivery	Micropulse train	Continuous-wave	Microsecond pulse train
Common wavelengths	577 nm, although 810 nm and other wavelengths have been reported	577 nm	527 nm; other wavelengths have been investigated
Typical spot size	100–200 μm; protocol-dependent	Protocol-dependent; not specified here	200 μm
Pulse duration	Typically 0.1–0.3 s per treatment envelope	Continuous-wave exposure; duration protocol-dependent	1.7 μs per micropulse
Pulse number/duty cycle	Repeated micropulses; typically 5% duty cycle	Not applicable (continuous-wave delivery)	Typically 3–30 micropulses per spot; protocol-dependent
Intended RPE response	Sublethal RPE modulation	Sublethal photothermal RPE stimulation	Selective RPE injury followed by repair/repopulation
Visibility of treatment spots	None intended	None intended	None immediately visible; delayed subtle fundus changes may occur
Dosimetry/titration	Threshold-based test-spot titration with subsequent power reduction; fixed-parameter protocols have also been used	Barely visible test-spot threshold followed by algorithm-based energy reduction using EpM	Individualized dosimetry/titration using FA-based, optoacoustic, reflectometric feedback-controlled, or fundus image-based approaches; FA-positive responses have been reported with 3 micropulses test exposures.
Primary treatment area in CSC	Macular areas affected by SRF and/or RPE dysfunction; broader macular coverage in high-density protocols	Patterned treatment over the involved macular region	FA-defined RPE leakage
Relationship to leakage site	Generally not restricted to individual leakage sites	Standard protocol not restricted to individual leakage sites; modified leakage-directed approaches reported	Leakage-directed
Treatment pattern	Grid/pattern-based treatment, including high-density or confluent macular coverage; protocol-dependent	Pattern-based	Approximately one-spot spacing
Treatment area with focal leakage	May extend beyond the focal leakage area	May extend beyond the focal leakage area	Primarily directed to the focal leakage area
Treatment area with diffuse leakage	Variable; broader affected-area treatment may be used	Broader pattern-based treatment	Broader treatment following the distribution of leakage
Foveal treatment	Protocol-dependent	Protocol-dependent	Leakage-directed; may include foveal leakage when indicated
Key technical consideration	Protocol heterogeneity and uncertainty in subthreshold dosimetry	Accuracy of individual threshold determination and algorithm-based reduction	Reliable, individualized determination of selective RPE injury

Abbreviations: CSC, central serous chorioretinopathy; EpM, Endpoint Management; FA, fluorescein angiography; NRT, nondamaging retinal laser therapy; RPE, retinal pigment epithelium; SMPL, subthreshold micropulse laser; SRF, subretinal fluid; SRT, selective retina therapy.

## Data Availability

No new data were created or analyzed in this study. Data sharing is not applicable to this article.

## Abbreviations

The following abbreviations are used in this manuscript:

BCVA	best-corrected visual acuity
CSC	central serous chorioretinopathy
ETDRS	Early Treatment Diabetic Retinopathy Study
EpM	Endpoint Management
FA	fluorescein angiography
FAF	fundus autofluorescence
HSP	heat-shock protein
NRT	nondamaging retinal laser therapy
OCT	optical coherence tomography
PDT	photodynamic therapy
PED	pigment epithelial detachment
RPE	retinal pigment epithelium
SMPL	subthreshold micropulse laser
SRF	subretinal fluid
SRT	selective retina therapy

## References

[1] Kitzmann A.S., Pulido J.S., Diehl N.N., Hodge D.O., Burke J.P. (2008). The incidence of central serous chorioretinopathy in Olmsted County, Minnesota, 1980–2002. Ophthalmology.

[2] Daruich A., Matet A., Dirani A., Bousquet E., Zhao M., Farman N., Jaisser F., Behar-Cohen F. (2015). Central serous chorioretinopathy: Recent findings and new physiopathology hypothesis. Prog. Retin. Eye Res..

[3] Feenstra H.M.A., van Dijk E.H.C., Cheung C.M.G., Ohno-Matsui K., Lai T.Y.Y., Koizumi H., Larsen M., Querques G., Downes S.M., Yzer S. (2024). Central serous chorioretinopathy: An evidence-based treatment guideline. Prog. Retin. Eye Res..

[4] Leaver P., Williams C. (1979). Argon laser photocoagulation in the treatment of central serous retinopathy. Br. J. Ophthalmol..

[5] Robertson D.M., Ilstrup D. (1983). Direct, indirect, and sham laser photocoagulation in the management of central serous chorioretinopathy. Am. J. Ophthalmol..

[6] Nicholson B., Noble J., Forooghian F., Meyerle C. (2013). Central serous chorioretinopathy: Update on pathophysiology and treatment. Surv. Ophthalmol..

[7] Ficker L., Vafidis G., While A., Leaver P. (1988). Long-term follow-up of a prospective trial of argon laser photocoagulation in the treatment of central serous retinopathy. Br. J. Ophthalmol..

[8] Chhablani J., Roh Y.J., Jobling A.I., Fletcher E.L., Lek J.J., Bansal P., Guymer R., Luttrull J.K. (2018). Restorative retinal laser therapy: Present state and future directions. Surv. Ophthalmol..

[9] Lanzetta P., Furlan F., Morgante L., Veritti D., Bandello F. (2008). Nonvisible subthreshold micropulse diode laser (810 nm) treatment of central serous chorioretinopathy. A pilot study. Eur. J. Ophthalmol..

[10] Sim S.Y., Lim L.T., Chaudhary S., Musa M., Hamilton R., Nicholson L., Pal B., Saini P., Mankongcharoen P., Sen S. (2026). Update on subthreshold micropulse laser treatment for retinal diseases: A narrative review. Surv. Ophthalmol..

[11] Scholz P., Altay L., Fauser S. (2017). A Review of Subthreshold Micropulse Laser for Treatment of Macular Disorders. Adv. Ther..

[12] Sramek C., Mackanos M., Spitler R., Leung L.S., Nomoto H., Contag C.H., Palanker D. (2011). Non-damaging retinal phototherapy: Dynamic range of heat shock protein expression. Investig. Ophthalmol. Vis. Sci..

[13] Lavinsky D., Sramek C., Wang J., Huie P., Dalal R., Mandel Y., Palanker D. (2014). Subvisible retinal laser therapy: Titration algorithm and tissue response. Retina.

[14] Lavinsky D., Palanker D. (2015). Nondamaging photothermal therapy for the retina: Initial clinical experience with chronic central serous retinopathy. Retina.

[15] Roider J., Michaud N.A., Flotte T.J., Birngruber R. (1992). Response of the retinal pigment epithelium to selective photocoagulation. Arch. Ophthalmol..

[16] Roider J., Hillenkamp F., Flotte T., Birngruber R. (1993). Microphotocoagulation: Selective effects of repetitive short laser pulses. Proc. Natl. Acad. Sci. USA.

[17] Brinkmann R., Roider J., Birngruber R. (2006). Selective retina therapy (SRT): A review on methods, techniques, preclinical and first clinical results. Bull. Soc. Belge. Ophtalmol..

[18] Fossataro F., Fossataro C., Abraham N., Fouad Y., Mrejen S., Tan A.C.S., Voichanski S., Sarraf D. (2024). Pathogenesis of Central Serous Chorioretinopathy and the Link Between Choroidal Hyperpermeability and Retinal Pigment Epithelium Pump Reversal. Am. J. Ophthalmol..

[19] Kim L.A., Maguire M.G., Weng C.Y., Smith J.R., Jain N., Flaxel C.J., Patel S., Kim S.J., Yeh S. (2025). Therapies for Central Serous Chorioretinopathy: A Report by the American Academy of Ophthalmology. Ophthalmology.

[20] van Dijk E.H.C., Fauser S., Breukink M.B., Blanco-Garavito R., Groenewoud J.M.M., Keunen J.E.E., Peters P.J.H., Dijkman G., Souied E.H., MacLaren R.E. (2018). Half-Dose Photodynamic Therapy versus High-Density Subthreshold Micropulse Laser Treatment in Patients with Chronic Central Serous Chorioretinopathy: The PLACE Trial. Ophthalmology.

[21] van Rijssen T.J., van Dijk E.H.C., Tsonaka R., Feenstra H.M.A., Dijkman G., Peters P.J.H., Diederen R.M.H., Hoyng C.B., Schlingemann R.O., Boon C.J.F. (2022). Half-Dose Photodynamic Therapy Versus Eplerenone in Chronic Central Serous Chorioretinopathy (SPECTRA): A Randomized Controlled Trial. Am. J. Ophthalmol..

[22] Lange C.A., Qureshi R., Pauleikhoff L. (2025). Interventions for central serous chorioretinopathy: A network meta-analysis. Cochrane Database Syst. Rev..

[23] Spitznas M. (1986). Pathogenesis of central serous retinopathy: A new working hypothesis. Graefes Arch. Clin. Exp. Ophthalmol..

[24] Frambach D.A., Marmor M.F. (1982). The rate and route of fluid resorption from the subretinal space of the rabbit. Investig. Ophthalmol. Vis. Sci..

[25] Marmor M.F. (1990). Control of subretinal fluid: Experimental and clinical studies. Eye.

[26] Yannuzzi L.A., Slakter J.S., Gross N.E., Spaide R.F., Costa D., Huang S.J., Klancnik J.M., Aizman A. (2003). Indocyanine green angiography-guided photodynamic therapy for treatment of chronic central serous chorioretinopathy: A pilot study. Retina.

[27] Pauleikhoff L.J.B., Diederen R.M.H., Chang-Wolf J.M., Moll A.C., Schlingemann R.O., van Dijk E.H.C., Boon C.J.F. (2024). Choroidal hyperpermeability patterns correlate with disease severity in central serous chorioretinopathy: CERTAIN study report 2. Acta Ophthalmol..

[28] Venkatesh R., Hande P., Prabhu V., Sahoo N.K., Hasan N., Chhablani J. (2026). The “Tap-Drain” model of central serous chorioretinopathy: Mechanistic staging and a corrective action-preventative action-based framework for disease modification. Surv. Ophthalmol..

[29] Brinkmann R., Hüttmann G., Rögener J., Roider J., Birngruber R., Lin C.P. (2000). Origin of retinal pigment epithelium cell damage by pulsed laser irradiance in the nanosecond to microsecond time regimen. Lasers Surg. Med..

[30] Hirabayashi K., Kakihara S., Tanaka M., Shindo T., Murata T. (2020). Investigation of the therapeutic mechanism of subthreshold micropulse laser irradiation in retina. Graefes Arch. Clin. Exp. Ophthalmol..

[31] Shiraya T., Araki F., Nakagawa S., Ueta T., Totsuka K., Abe H., Naito Y., Toyama T., Sugimoto K., Kato S. (2022). Differential gene expression analysis using RNA sequencing: Retinal pigment epithelial cells after exposure to continuous-wave and subthreshold micropulse laser. Jpn. J. Ophthalmol..

[32] De Cillà S., Vezzola D., Farruggio S., Vujosevic S., Clemente N., Raina G., Mary D., Casini G., Rossetti L., Avagliano L. (2019). The subthreshold micropulse laser treatment of the retina restores the oxidant/antioxidant balance and counteracts programmed forms of cell death in the mice eyes. Acta Ophthalmol..

[33] Midena E., Bini S., Martini F., Enrica C., Pilotto E., Micera A., Esposito G., Vujosevic S. (2020). Changes of aqueous humor müller cells’ biomarkers in human patients affected by diabetic macular edema after subthreshold micropulse laser treatment. Retina.

[34] Luttrull J.K. (2016). Low-intensity/high-density subthreshold diode micropulse laser for central serous chorioretinopathy. Retina.

[35] Lavinsky D., Wang J., Huie P., Dalal R., Lee S.J., Lee D.Y., Palanker D. (2016). Nondamaging Retinal Laser Therapy: Rationale and Applications to the Macula. Investig. Ophthalmol. Vis. Sci..

[36] Framme C., Schuele G., Roider J., Birngruber R., Brinkmann R. (2004). Influence of pulse duration and pulse number in selective RPE laser treatment. Lasers Surg. Med..

[37] Kretz F.T., Beger I., Koch F., Nowomiejska K., Auffarth G.U., Koss M.J. (2015). Randomized Clinical Trial to Compare Micropulse Photocoagulation Versus Half-dose Verteporfin Photodynamic Therapy in the Treatment of Central Serous Chorioretinopathy. Ophthalmic Surg. Lasers Imaging Retin..

[38] Arora S., Sridharan P., Arora T., Chhabra M., Ghosh B. (2019). Subthreshold diode micropulse laser versus observation in acute central serous chorioretinopathy. Clin. Exp. Optom..

[39] Sun Z., Huang Y., Nie C., Wang Z., Pei J., Lin B., Zhou R., Zhang J., Chong V., Liu X. (2020). Efficacy and safety of subthreshold micropulse laser compared with threshold conventional laser in central serous chorioretinopathy. Eye.

[40] Zhou L., Lai K., Jin L., Huang C., Xu F., Gong Y., Li L., Zhu Z., Lu L., Jin C. (2021). Subthreshold Micropulse Laser vs. Conventional Laser for Central Serous Chorioretinopathy: A Randomized Controlled Clinical Trial. Front. Med..

[41] Zhou L., Huang C., Li Z., Lu T., Zhao H., Li C., Cong Q., Liang J., Zhong X., Jin L. (2024). A randomized non-inferiority trial of 577nm subthreshold micropulse laser versus half-dose photodynamic therapy for acute central serous chorioretinopathy. Photodiagnosis Photodyn. Ther..

[42] Brelen M.E., Ho M., Li S., Ng D.S.C., Yip Y.W.Y., Lee W.S., Chen L.J., Young A.L., Tham C.C., Pang C.P. (2024). Comparing Half-Dose Photodynamic Therapy with Subthreshold Micropulse Laser for the Treatment of Central Serous Chorioretinopathy. Ophthalmol. Retin..

[43] Ho M., Lai F.H.P., Ng D.S.C., Iu L.P.L., Chen L.J., Mak A.C.Y., Yip Y., Cheung C., Young A.L., Brelen M. (2021). Analysis of choriocapillaris perfusion and choroidal layer changes in patients with chronic central serous chorioretinopathy randomised to micropulse laser or photodynamic therapy. Br. J. Ophthalmol..

[44] Wood E.H., Karth P.A., Sanislo S.R., Moshfeghi D.M., Palanker D.V. (2017). Nondamaging retinal laser therapy for treatment of central serous chorioretinopathy: What is the evidence?. Retina.

[45] Klatt C., Saeger M., Oppermann T., Pörksen E., Treumer F., Hillenkamp J., Fritzer E., Brinkmann R., Birngruber R., Roider J. (2011). Selective retina therapy for acute central serous chorioretinopathy. Br. J. Ophthalmol..

[46] Framme C., Walter A., Berger L., Prahs P., Alt C., Theisen-Kunde D., Kowal J., Brinkmann R. (2015). Selective Retina Therapy in Acute and Chronic-Recurrent Central Serous Chorioretinopathy. Ophthalmologica.

[47] Oh J., Yoon C.K., Kim B.H., Yu H.G. (2021). Evaluation of the Safety and Efficacy of Selective Retina Therapy Laser Treatment in Patients with Central Serous Chorioretinopathy. Korean J. Ophthalmol..

[48] Lee J.Y., Kim M.H., Jeon S.H., Lee S.H., Roh Y.J. (2021). The Effect of Selective Retina Therapy with Automatic Real-Time Feedback-Controlled Dosimetry for Chronic Central Serous Chorioretinopathy: A Randomized, Open-Label, Controlled Clinical Trial. J. Clin. Med..

[49] Büttner M., Luger B., Abou Moulig W., Junker B., Framme C., Jacobsen C., Knoll K., Pielen A. (2021). Selective retina therapy (SRT) in patients with therapy refractory persistent acute central serous chorioretinopathy (CSC): 3 months functional and morphological results. Graefes Arch. Clin. Exp. Ophthalmol..

[50] Yoon C.K., Yu H.G. (2024). Selective retina therapy with real-time feedback-controlled technology in central serous chorioretinopathy: A 24-month follow-up real-world prospective study. BMJ Open Ophthalmol..

[51] Jeon S.H., Kim D., Roh Y.J. (2026). Selective Retina Therapy With Fundus Image-Based Titration in Central Serous Chorioretinopathy: Prospective Outcomes and Predictive Factors. Transl. Vis. Sci. Technol..

[52] Schuele G., Elsner H., Framme C., Roider J., Birngruber R., Brinkmann R. (2005). Optoacoustic real-time dosimetry for selective retina treatment. J. BioMed Opt..

[53] Park Y.G., Seifert E., Roh Y.J., Theisen-Kunde D., Kang S., Brinkmann R. (2014). Tissue response of selective retina therapy by means of a feedback-controlled energy ramping mode. Clin. Exp. Ophthalmol..

[54] Jeon S.H., Kim M., Roh Y.J. (2021). Retinal pigment epithelial responses based on the irradiation density of selective retina therapy. Graefes Arch. Clin. Exp. Ophthalmol..

[55] Jeon S.H., Kim M., Roh Y.J. (2024). Use of a Fundus Image-Based Titration Strategy for Selective Retina Therapy for Central Serous Chorioretinopathy. J. Clin. Med..

[56] Banerjee R.K., Zhu L., Gopalakrishnan P., Kazmierczak M.J. (2007). Influence of laser parameters on selective retinal treatment using single-phase heat transfer analyses. Med. Phys..

[57] Kim D., Jeon S.H., Roh Y.J. (2026). Effects of Selective Retina Therapy on Central Serous Chorioretinopathy with Serous Pigment Epithelial Detachments. J. Clin. Med..

[58] van Rijssen T.J., van Dijk E.H.C., Scholz P., Breukink M.B., Dijkman G., Peters P.J.H., Tsonaka R., Keunen J.E.E., MacLaren R.E., Hoyng C.B. (2021). Long-term follow-up of chronic central serous chorioretinopathy after successful treatment with photodynamic therapy or micropulse laser. Acta Ophthalmol..

[59] Scholz P., Altay L., Fauser S. (2016). Comparison of subthreshold micropulse laser (577 nm) treatment and half-dose photodynamic therapy in patients with chronic central serous chorioretinopathy. Eye.

[60] Schworm B., Siedlecki J., Keidel L.F., Herold T.R., Luft N., Priglinger S.G. (2021). Subthreshold laser therapy with a standardized macular treatment pattern in chronic central serous chorioretinopathy. Graefes Arch. Clin. Exp. Ophthalmol..

[61] Kaderli S.T., Karalezli A., Kaderli A., Taskin S.C., Sul S. (2022). Evaluation of the choroidal vascularity index after subthreshold yellow laser treatment in the patients with chronic central serous chorioretinopathy. Eye.

[62] Lee S.H., Lee J., Kim M., Roh Y.J. (2024). The effect of nondamaging subthreshold laser therapy in patients with chronic central serous chorioretinopathy. Graefes Arch. Clin. Exp. Ophthalmol..

[63] Önen M., Zor K.R., Doğan L., Küçük E., Yıldırım Biçer G., Özer Ö. (2025). The results of subthreshold 577 nm yellow laser application using pascal laser system in patients with chronic central serous chorioretinopathy. Lasers Med. Sci..

[64] Elsner H., Pörksen E., Klatt C., Bunse A., Theisen-Kunde D., Brinkmann R., Birngruber R., Laqua H., Roider J. (2006). Selective retina therapy in patients with central serous chorioretinopathy. Graefes Arch. Clin. Exp. Ophthalmol..

[65] Kim Y.J., Lee Y.G., Lee D.W., Kim J.H. (2018). Selective Retina Therapy with Real-Time Feedback-Controlled Dosimetry for Treating Acute Idiopathic Central Serous Chorioretinopathy in Korean Patients. J. Ophthalmol..

[66] Yadav N.K., Jayadev C., Mohan A., Vijayan P., Battu R., Dabir S., Shetty B., Shetty R. (2015). Subthreshold micropulse yellow laser (577 nm) in chronic central serous chorioretinopathy: Safety profile and treatment outcome. Eye.

[67] Kiraly P., Habjan M., Smrekar J., Mekjavić P.J. (2023). Functional Outcomes and Safety Profile of Trans-Foveal Subthreshold Micropulse Laser in Persistent Central Serous Chorioretinopathy. Life.

[68] Chhablani J., Kalra G., Alkwatli L., Fassbender B., Amoroso F., Chandra K., Ankireddy S., Maltsev D., Striebe N.A., Souied E. (2021). Safety of various parameter sets with navigated microsecond pulsing laser in central serous chorioretinopathy. Int. J. Retin. Vitr..

[69] Enríquez-Fuentes J.E., Alarcón-García A.D., Oribio-Quinto C., Fernández-Vigo J.I. (2023). Hyperplasia of the retinal pigment epithelium secondary to subthreshold laser treatment in chronic central serous chorioretinopathy. Photodiagnosis Photodyn. Ther..

[70] Gawęcki M. (2015). Increase in Central Retinal Edema after Subthreshold Diode Micropulse Laser Treatment of Chronic Central Serous Chorioretinopathy. Case Rep. Ophthalmol. Med..

[71] Yasui A., Yamamoto M., Hirayama K., Shiraki K., Theisen-Kunde D., Brinkmann R., Miura Y., Kohno T. (2017). Retinal sensitivity after selective retina therapy (SRT) on patients with central serous chorioretinopathy. Graefes Arch. Clin. Exp. Ophthalmol..

[72] Jeon S.H., Kim M., Lee J., Roh Y.J. (2022). The Effect of Selective Retina Therapy for Bevacizumab-Resistant Chronic Central Serous Chorioretinopathy. Ophthalmologica.

[73] Lindziute M., Binter M., Al Mahamed N., Pielen A., Framme C., Tode J. (2026). Long-term outcomes of titration-guided focal selective retina therapy for chronic central serous chorioretinopathy: Retrospective real-world data analysis. BMC Ophthalmol..

[74] Pollack J.S., Kim J.E., Pulido J.S., Burke J.M. (1998). Tissue effects of subclinical diode laser treatment of the retina. Arch. Ophthalmol..

[75] Yu A.K., Merrill K.D., Truong S.N., Forward K.M., Morse L.S., Telander D.G. (2013). The comparative histologic effects of subthreshold 532- and 810-nm diode micropulse laser on the retina. Investig. Ophthalmol. Vis. Sci..

[76] Yang J.H., Yu S.Y., Kim T.G., Kim E.S., Kwak H.W. (2016). Morphologic changes in the retina after selective retina therapy. Graefes Arch. Clin. Exp. Ophthalmol..

[77] Framme C., Brinkmann R., Birngruber R., Roider J. (2002). Autofluorescence imaging after selective RPE laser treatment in macular diseases and clinical outcome: A pilot study. Br. J. Ophthalmol..

[78] Binter M., Lindziute M., Rosenstein C., Framme C., Tode J. (2023). Long-Term Multimodal Imaging Analysis of Selective Retina Therapy Laser Lesions. Life.

[79] Treumer F., Klettner A., Baltz J., Hussain A.A., Miura Y., Brinkmann R., Roider J., Hillenkamp J. (2012). Vectorial release of matrix metalloproteinases (MMPs) from porcine RPE-choroid explants following selective retina therapy (SRT): Towards slowing the macular ageing process. Exp. Eye Res..

[80] Richert E., Koinzer S., Tode J., Schlott K., Brinkmann R., Hillenkamp J., Klettner A., Roider J. (2018). Release of Different Cell Mediators During Retinal Pigment Epithelium Regeneration Following Selective Retina Therapy. Investig. Ophthalmol. Vis. Sci..

[81] Kyo A., Yamamoto M., Hirayama K., Kohno T., Theisen-Kunde D., Brinkmann R., Miura Y., Honda S. (2021). Factors affecting resolution of subretinal fluid after selective retina therapy for central serous chorioretinopathy. Sci. Rep..

[82] Kim M., Jeon S.H., Lee J.Y., Lee S.H., Roh Y.J. (2022). Factors Predicting Response to Selective Retina Therapy in Patients with Chronic Central Serous Chorioretinopathy. J. Clin. Med..

[83] Marmor M.F. (1988). New hypotheses on the pathogenesis and treatment of serous retinal detachment. Graefes Arch. Clin. Exp. Ophthalmol..

[84] Mrejen S., Sarraf D., Mukkamala S.K., Freund K.B. (2013). Multimodal imaging of pigment epithelial detachment: A guide to evaluation. Retina.

[85] Loo R.H., Scott I.U., Flynn H.W., Gass J.D., Murray T.G., Lewis M.L., Rosenfeld P.J., Smiddy W.E. (2002). Factors associated with reduced visual acuity during long-term follow-up of patients with idiopathic central serous chorioretinopathy. Retina.

[86] Mudvari S.S., Goff M.J., Fu A.D., McDonald H.R., Johnson R.N., Ai E., Jumper J.M. (2007). The natural history of pigment epithelial detachment associated with central serous chorioretinopathy. Retina.

[87] Feenstra H.M.A., Hahn L.C., van Rijssen T.J., Tsonaka R., Breukink M.B., Keunen J.E.E., Peters P.J.H., Dijkman G., Souied E.H., MacLaren R.E. (2022). Efficacy of half-dose photodynamic therapy versus high-density subthreshold micropulse laser for treating pigment epithelial detachments in chronic central serous chorioretinopathy. Retina.

[88] Liu L., Xu Q., Song X., Zhao Y., Zhang Y., Qu Y. (2024). Evaluation of changes in macular structures after subthreshold micropulse laser therapy on chronic central serous chorioretinopathy. Lasers Med. Sci..

[89] Klatt C., Elsner H., Pörksen E., Brinkmann R., Bunse A., Birngruber R., Roider J. (2006). Selective retina therapy in central serous chorioretinopathy with detachment of the pigmentary epithelium. Ophthalmologe.

[90] Park Y.G., Kang S., Kim M., Yoo N., Roh Y.J. (2017). Selective retina therapy with automatic real-time feedback-controlled dosimetry for chronic central serous chorioretinopathy in Korean patients. Graefes Arch. Clin. Exp. Ophthalmol..

[91] Yamada-Okahara N., Kyo A., Hirayama K., Yamamoto M., Kohno T., Honda S. (2023). Practical treatment options for persistent central serous chorioretinopathy and early visual and anatomical outcomes. Jpn. J. Ophthalmol..

[92] Arsan A., Kanar H.S., Sonmez A. (2018). Visual outcomes and anatomic changes after sub-threshold micropulse yellow laser (577-nm) treatment for chronic central serous chorioretinopathy: Long-term follow-up. Eye.

[93] Maruko I., Koizumi H., Hasegawa T., Arakawa H., Iida T. (2017). Subthreshold 577 nm micropulse laser treatment for central serous chorioretinopathy. PLoS ONE.

[94] Beniwal A., Shaikh N., Chawla R., Azad S.V., Kumar V., Vohra R. (2022). Comparison of two protocols of subthreshold micropulse yellow laser treatment for non-resolving central serous chorioretinopathy. Indian J. Ophthalmol..

[95] von der Burchard C., Miura Y., Stanzel B., Chhablani J., Roider J., Framme C., Brinkmann R., Tode J. (2024). Regenerative Retinal Laser and Light Therapies (RELITE): Proposal of a New Nomenclature, Categorization, and Trial Reporting Standard. Lasers Surg. Med..

[96] Chen S.N., Hwang J.F., Tseng L.F., Lin C.J. (2008). Subthreshold diode micropulse photocoagulation for the treatment of chronic central serous chorioretinopathy with juxtafoveal leakage. Ophthalmology.

[97] van Rijssen T.J., van Dijk E.H.C., Scholz P., Breukink M.B., Blanco-Garavito R., Souied E.H., Keunen J.E.E., MacLaren R.E., Querques G., Fauser S. (2019). Focal and Diffuse Chronic Central Serous Chorioretinopathy Treated With Half-Dose Photodynamic Therapy or Subthreshold Micropulse Laser: PLACE Trial Report No. 3. Am J Ophthalmol..

[98] Chhablani J., Cohen F.B. (2020). Multimodal Imaging-Based Central Serous Chorioretinopathy Classification. Ophthalmol. Retin..

[99] Getahun H., Apte R.S. (2025). Therapeutic interventions for chronic central serous chorioretinopathy: A comprehensive assessment of systematic reviews. Int. J. Retin. Vitr..

